# Amazonian Plants: A Global Bibliometric Approach to *Petiveria alliacea* L. Pharmacological and Toxicological Properties

**DOI:** 10.3390/plants12183343

**Published:** 2023-09-21

**Authors:** Brenda Costa da Conceição, Thales Andrade da Silva, Lucas Villar Pedrosa da Silva Pantoja, Diandra Araújo da Luz, Eloise Karoline Serrão Cardoso, Laryssa Danielle da Silva Reis, Maria Carolina Raiol-da-Silva, Monique Silva Kussler, Cristiane Socorro Ferraz Maia, Enéas Andrade Fontes-Júnior

**Affiliations:** Laboratory of Pharmacology of Inflammation and Behavior, Faculty of Pharmacy, Institute of Health Sciences, Federal University of Pará, Belém 66075-110, PA, Brazil; breuscsta@gmail.com (B.C.d.C.); thalessilva@ics.ufpa.br (T.A.d.S.); lucas-villar@outlook.com (L.V.P.d.S.P.); diandra.luz@ics.ufpa.br (D.A.d.L.); serraoeloise@gmail.com (E.K.S.C.); laryssadreis@gmail.com (L.D.d.S.R.); m.carolraiol@gmail.com (M.C.R.-d.-S.); monique.kussler@ics.ufpa.br (M.S.K.); crismaia@ufpa.br (C.S.F.M.)

**Keywords:** *Petiveria alliacea*, medicinal plants, bibliometric analysis, toxicology, phamacological properties

## Abstract

*Petiveria alliacea* L. (Phytolaccaceae) holds significant importance in the Amazon region, where it has been traditionally utilized in folk medicine. In this study, we conducted a comprehensive bibliometric analysis using conventional metrics, combined with a critical content review of its pharmacological and toxicological properties, to identify gaps in the existing literature that require further investigation. Our investigation identified a total of 55 articles that met the inclusion criteria for this study. Remarkably, Brazil emerged as the primary contributor within the scope of this review, indicating a strong presence of research from this country. Furthermore, professional scientific societies have played a pivotal role in facilitating the dissemination of scientific findings through specialist journals, fostering the sharing of research work within the community. Analysis of keyword co-occurrence revealed that “*Petiveria alliacea*”, “plant extract”, and “guatemala” were the most frequently encountered terms, indicating their significance within the literature. In terms of study designs, in vivo and in vitro were the predominant types observed, highlighting their prevalence in this field of study. Our study also identified a lack in knowledge yet to be investigated.

## 1. Introduction

Medicinal plants are renowned for their traditional application in the treatment of diseases due to their therapeutic properties, being present in the most diverse cultures and civilizations throughout the ages [1]. Even today, these plant species are widely sought after due to the expectation of lower risks of adverse reactions and toxicity, which is associated with their natural origin, coupled with easy access and low cost. For similar reasons, they have become the main source of new drug development [2]. In this context, the Amazon biome, which includes one of the largest tropical forests in the world, represents a rich storehouse of species and biomolecules whose therapeutic applicability has been the target of numerous studies validating or reforming folk medicine [3,4,5].

*Petiveria alliacea* L. (Phytolaccaceae) is a plant species widely distributed in the Amazon region but also found in various regions of the Americas, including the Caribbean islands and Mexico [6]. It is characterized as an herbaceous plant with a height ranging from 5 to 150 cm, featuring an erect, cylindrical stem and branches [7]. In traditional medicine, *P. alliacea* finds application in various therapeutic approaches, among which stand out antispasmodic, diuretic, abortive, hypoglycemic, anti-inflammatory, and anticancer activity, in addition to central nervous system activities, such as anxiolytic effects, and use in the treatment of dementia and nervous disorders [8]. Pre-clinical studies on the species, aimed at clarifying the safety of its use and verifying the properties attributed to it by popular knowledge, have validated some of its properties, offering initial information that subsidizes its rational use, as well as the development of herbal medicines and medicines based on their derivatives [9,10,11,12].

Given the different models, mechanisms, and applications already investigated, reviewing this content is important to synthesize discoveries, connect conclusions, and identify gaps in knowledge to be filled. Bibliometric analysis, in turn, emerges as a type of study that utilizes quantitative measures to evaluate the influence, visibility and impact of research output [13]. Overall, these analyses contribute to assessing the impact and trends within a specific field, supporting evidence-based decision-making in research [14].

This study aimed to conduct comprehensive global research using bibliometric analysis to evaluate the scientific production concerning the pharmacological and toxicological activities of *P. alliacea* L. The objective was to collect relevant metric data and provide a global perspective on key aspects, such as study designs, notable authors, contributing countries, and more. By employing bibliometric analysis, the study sought to identify trends, patterns, and research gaps within the existing literature on *P. alliacea*. Additionally, the study aimed to highlight areas within the literature that require further investigation and exploration, thereby facilitating the identification of research opportunities and potential future directions in this field. 

## 2. Results 

### 2.1. Bibliometric Analysis

**Figure 1:**
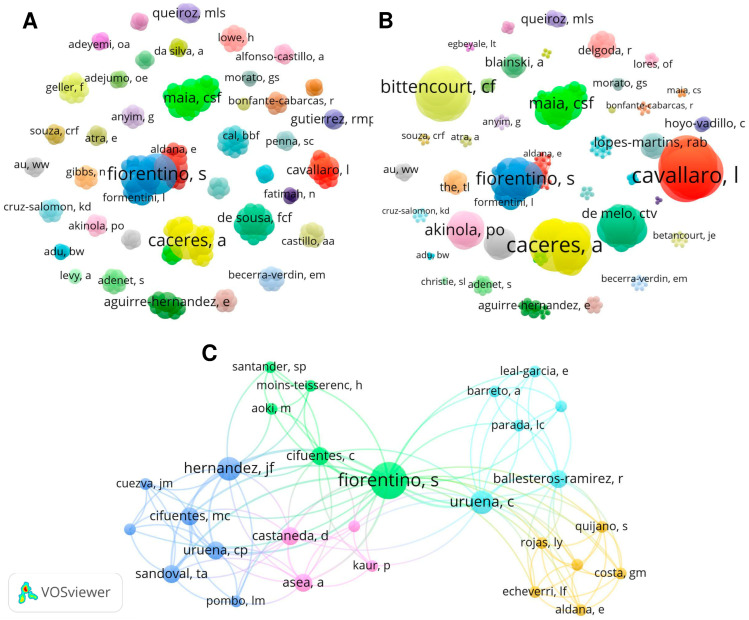
Network visualization of authors with the number of publications (**A**), citations (**B**), and leading network of authors (**C**). There is a direct proportionality of the cluster size and the number of publications or citations.

Through the search query performed in WoS-CC, a total of 174 articles was retrieved, of which 55 were selected (Table 1) after meeting the inclusion criteria (Figure 1). The Supplementary Section presents the excluded papers along with the reasons for exclusion.

The oldest of the selected papers was published in 1991 [15,16]. Interestingly, both articles reported the analgesic properties of *P. alliacea*. Using a murine model, de Lima et al. [16] confirmed the analgesic effect of *P. alliacea*, which was already used in many Latin American and African countries for pain relief. In addition, Ferraz et al. [15], in a more robust research protocol (randomized clinical trial), also reported the analgesic activity of *P. alliacea* in patients with osteoarthritis. The most recent paper was published in 2023 and addressed the cytotoxic properties of *P. alliacea* against tumor cells [17]. The most cited article is a pre-clinical (in vitro) study that also investigated the cytotoxic effects of *P. alliacea* against hepatic cancer cells [18].

**Table 1:** Selected articles on *Petiveria alliacea* L. in WoS-CC.

Authors/Year	Article Title	DOI/URL (Accessed on 3 August 2023)	Number of Citations		
			WoS-CC	Scopus	Google Scholar
Prada et al., 2023 [17]	Doxorubicin Activity Is Modulated by Traditional Herbal Extracts in a 2D and 3D Multicellular Sphere Model of Leukemia	https://doi.org//10.3390/pharmaceutics15061690	0	0	0
Cal et al., 2022 [6]	Cytotoxicity of Extracts from *Petiveria alliacea* Leaves on Yeast	https://doi.org//10.3390/plants11233263	0	0	1
Cruz-Salomon et al., 2022 [8]	In vivo and In Silico Study of the Antinociceptive and Toxicological Effect of the Extracts of *Petiveria alliacea* L. Leaves	https://doi.org//10.3390/ph15080943	0	0	1
Olajubutu et al., 2022 [19]	Topical Anti-Inflammatory Activity of *Petiveria alliacea*, Chemical Profiling and Computational Investigation of Phytoconstituents Identified from its Active Fraction	https://doi.org//10.1007/s42250-022-00363-y	4	0	0
Zavala-Ocampo et al., 2022 [2]	Acetylcholinesterase inhibition and antioxidant activity properties of *Petiveria alliacea* L.	https://doi.org//10.1016/j.jep.2022.115239	5	6	7
Castaneda et al., 2022 [12]	Medicinal plants used in traditional Mayan medicine for the treatment of central nervous system disorders: An overview	https://doi.org//10.1016/j.jep.2021.114746	7	7	8
Mustika et al., 2021 [4]	The self-nanoemulsifying drug delivery system of *Petiveria alliacea* extract reduced the homeostatic model assessment-insulin resistance value, interleukin-6, and tumor necrosis factor-alpha level in diabetic rat models	https://doi.org//10.14202/vetworld.2021.3229-3234	0	0	1
Oyeleke et al., 2021 [7]	Antimicrobial, immunomodulatory and hepatomodulatory effects of aqueous extracts of *Petiveria alliacea* root and leaf on growing pullets	https://doi.org//10.5424/sjar/2021191-17300	1	1	2
Ileke et al., 2021 [9]	Bioefficacy of two indigenous Nigerian botanicals on the developmental stages of malaria vector, *Anopheles gambiaegiles* [Diptera: Culicidae]	https://doi.org//10.1007/s42690-020-00281-x	1	1	2
Ballesteros-Ramirez et al., 2020 [10]	Preferential Activity of *Petiveria alliacea* Extract on Primary Myeloid Leukemic Blast	https://doi.org//10.1155/2020/4736206	2	2	6
Caicedo-Pinto et al., 2019 [11]	Effects of *Petiveria alliacea* and cholinergic drugs on the habituation cognitive behavior	https://doi.org//10.35588/blacpma.19.18.6.42	1	1	1
Alves et al., 2019 [13]	*Petiveria alliacea*, a plant used in Afro-Brazilian smoke rituals, triggers pulmonary inflammation in rats	https://doi.org//10.1016/j.bjp.2019.06.005	4	5	9
Garcia-Perez et al., 2018 [20]	Toxicological evaluation of an aqueous suspension from leaves and stems of *Petiveria alliacea* L. (Phytolaccaceae)	https://doi.org//10.1016/j.jep.2017.09.022	7	9	18
Gutierrez and Flores, 2018 [14]	*Petiveria alliacea* Suppresses Airway Inflammation and Allergen-Specific Th2 Responses in Ovalbumin-Sensitized Murine Model of Asthma	https://doi.org//10.1007/s11655-018-2566-5	2	4	6
Lateef et al., 2018 [21]	Characterization, antimicrobial, antioxidant, and anticoagulant activities of silver nanoparticles synthesized from *Petiveria alliacea* L. Leaf extract	https://doi.org//10.1080/10826068.2018.1479864	63	75	100
Hartmann et al., 2018 [22]	Investigation of the Larvicidal Effect of Guinea (*Petiveria alliacea*) on Larvae of Mosquitoes of the Species *A. aegypti*	https://doi.org//10.21577/1984-6835.20180040	1	2	3
Flota-Burgos et al., 2017 [23]	Anthelminthic activity of methanol extracts of *Diospyros anisandra* and *Petiveria alliacea* on cyathostomin (Nematoda: Cyathostominae) larval development and egg hatching	https://doi.org/10.1016/j.vetpar.2017.10.016	7	9	16
Zavala-Ocampo et al., 2017 [24]	Antiamoebic Activity of *Petiveria alliacea* Leaves and Their Main Component, Isoarborinol	https://doi.org//10.4014/jmb.1705.05003	10	15	19
Hernandez et al., 2017 [25]	A cytotoxic *Petiveria alliacea* dry extract induces ATP depletion and decreases beta-F1-ATPase expression in breast cancer cells and promotes survival in tumor-bearing mice	https://doi.org//10.1016/j.bjp.2016.09.008	9	11	20
Gutierrez and Vadillo, 2017 [26]	Anti-inflammatory Potential of *Petiveria alliacea* on Activated RAW264.7 Murine Macrophages	https://doi.org//10.4103/pm.pm_479_16	20	22	28
Luz et al., 2016 [27]	Ethnobotany, phytochemistry and neuropharmacological effects of *Petiveria alliacea* L. (Phytolaccaceae): A review	https://doi.org//10.1016/j.jep.2016.02.053	35	45	70
Murray et al., 2016 [28]	Significant inhibitory impact of dibenzyl trisulfide and extracts of *Petiveria alliacea* on the activities of major drug-metabolizing enzymes in vitro: An assessment of the potential for medicinal plant-drug interactions	https://doi.org//10.1016/j.fitote.2016.04.011	19	19	25
Silva et al., 2015 [29]	*Petiveria alliacea* exerts mnemonic and learning effects on rats	https://doi.org//10.1016/j.jep.2015.04.005	11	12	21
Kerdudo et al., 2015 [30]	Essential oil composition and biological activities of *Petiveria alliacea* L. From Martinique	https://doi.org//10.1080/10412905.2015.1014118	9	11	13
Hernandez et al., 2014 [31]	A *Petiveria alliacea* standardized fraction induces breast adenocarcinoma cell death by modulating glycolytic metabolism	https://doi.org//10.1016/j.jep.2014.03.013	26	33	59
Christie and Levy, 2013 [32]	Evaluation of the Hypoglycaemic Activity of *Petiveria alliacea* (Guinea Hen Weed) Extracts in Normoglycaemic and Diabetic Rat Models	https://www.mona.uwi.edu/fms/wimj/system/files/article_pdfs/dr_christie_wimj_november.qxd_.pdf	6	7	13
Fletes-Arjona et al., 2013 [33]	Morphologic Alterations in the Respiratory Tract of Wistar Rats Induced by Steams of the Root of Hierba del Zorrillo (*Petiveria alliacea*) from Southwest Mexico	https://doi.org//10.4067/S0717-95022013000100019	3	3	1
Pacheco et al., 2013 [34]	In vitro antimicrobial activity of total extracts of the leaves of *Petiveria alliacea* L. (Anamu)	https://doi.org//10.1590/S1984-82502013000200006	8	9	19
de Andrade et al., 2012 [35]	Potential behavioral and pro-oxidant effects of *Petiveria alliacea* L. Extract in adult rats	https://doi.org//10.1016/j.jep.2012.07.020	16	20	33
Santander et al., 2012 [36]	Immunomodulatory Effects of Aqueous and Organic Fractions from *Petiveria alliacea* on Human Dendritic Cells	https://doi.org//10.1142/S0192415X12500620	11	13	23
Adejumo et al., 2011 [37]	Phytochemical and antisickling activities of *Entandrophragma utile*, *Chenopodium ambrosioides* and *Petiveria alliacea*	https://academicjournals.org/journal/JMPR	12	13	33
Duharte et al., 2011 [38]	Protecting effect of *Petiveria alliacea* (Anamu) on the immunosuppression induced by 5-fluorouracil in Balb/c mice	https://www.redalyc.org/pdf/856/85618379009.pdf	3	4	3
Maia et al., 2010 [39]	Analysis of Fetal and Placental Development in Rats after Administration of Hydroalcoholic Extract from the Root of *Petiveria alliacea* L. (Phytolaccaceae)	https://doi.org//10.4067/S0717-95022010000100023	2	2	5
Blainski et al., 2010 [40]	Dual effects of crude extracts obtained from *Petiveria alliacea* L. (Phytolaccaceae) on experimental anxiety in mice	https://doi.org/10.1016/j.jep.2010.01.012	30	32	56
Guedes et al., 2009 [41]	Antimicrobial activity of crude extracts of *Petiveria alliacea* L.	http://www.latamjpharm.org/trabajos/28/4/LAJOP_28_4_1_7_AIT6W1N9TT.pdf	3	8	2
Schmidt et al., 2009 [42]	Biological studies on Brazilian plants used in wound healing	https://doi.org//10.1016/j.jep.2009.01.022	92	101	194
Gomes et al., 2008 [43]	Central effects of isolated fractions from the root of *Petiveria alliacea* L. (tipi) in mice	https://doi.org//10.1016/j.jep.2008.08.012	35	39	59
Urueña et al., 2008 [44]	*Petiveria alliacea* extracts use multiple mechanisms to inhibit growth of human and mouse tumoral cells	https://doi.org//10.1186/1472-6882-8-60	38	46	92
Adomi, 2008 [45]	Screening of the leaves of three Nigerian medicinal plants for antibacterial activity	https://www.ajol.info/index.php/ajb/article/view/59084	8	9	33
Garcia-Gonzalez et al., 2006 [46]	Subchronic and acute preclinic toxicity and some pharmacological effects of the water extract from leaves of *Petiveria alliacea* (Phytolaccaceae)	https://doi.org//10.15517/rbt.v54i4.3108	9	11	18
Gomes et al., 2005 [47]	Study of antinociceptive effect of isolated fractions from *Petiveria alliacea* L. (tipi) in mice	https://doi.org//10.1248/bpb.28.42	29	34	64
Ruffa et al., 2002 [48]	Antiviral activity of *Petiveria alliacea* against the bovine viral diarrhea virus	https://doi.org//10.1159/000064920	10	12	28
Lopes-Martins et al., 2002 [49]	The anti-inflammatory and analgesic effects of a crude extract of *Petiveria alliacea* L. (Phytolaccaceae)	https://doi.org//10.1078/0944-7113-00118	32	46	94
Ruffa et al., 2002 [18]	Cytotoxic effect of Argentine medicinal plant extracts on human hepatocellular carcinoma cell line	https://doi.org//10.1016/S0378-8741(01)00400-7	149	171	302
Benevides et al., 2001 [50]	Antifungal polysulphides from *Petiveria alliacea* L.	https://doi.org//10.1016/S0031-9422(01)00079-6	50	62	114
Morales et al., 2001 [51]	Preliminary screening of five ethnomedicinal plants of Guatemala	https://doi.org//10.1016/S0014-827X(01)01107-7	11	13	21
Queiroz et al., 2000 [52]	Cytokine profile and natural killer cell activity in Listeria monocytogenes infected mice treated orally with *Petiveria alliacea* extract	https://doi.org//10.3109/08923970009026008	10	11	24
Quadros et al., 1999 [53]	*Petiveria alliacea* L. extract protects mice against Listeria monocytogenes infection-Effects on bone marrow progenitor cells	https://doi.org//10.3109/08923979909016397	15	16	35
Caceres et al., 1998 [54]	Plants used in Guatemala for the treatment of protozoal infections. I. Screening of activity to bacteria, fungi and American trypanosomes of 13 native plants	https://doi.org//10.1016/S0378-8741(98)00140-8	60	67	187
Berger et al., 1998 [55]	Plants used in Guatemala for the treatment of protozoal infections-II. Activity of extracts and fractions of five Guatemalan plants against *Trypanosoma cruzi*	https://doi.org//10.1016/S0378-8741(98)00011-7	42	48	100
Villar et al., 1997 [56]	Screening of 17 Guatemalan medicinal plants for platelet antiaggregant activity	https://doi.org//10.1002/(SICI)1099-1573(199709)11:6<441::AID-PTR126>3.0.CO;2-T	31	35	82
Williams et al., 1997 [57]	Immunomodulatory activities of *Petiveria alliacea* L.	https://doi.org//10.1002/(SICI)1099-1573(199705)11:3<251::AID-PTR75>3.0.CO;2-B	19	24	45
Hoyos et al., 1992 [58]	Evaluation of the genotoxic effects of a folk medicine, *Petiveria alliacea* (Anamu)	https://doi.org//10.1016/0165-1218(92)90015-r	14	15	48
de Lima et al., 1991 [16]	Evaluation of antinociceptive effect of *Petiveria alliacea* (guine) in animals	https://doi.org//10.1590/S0074-02761991000600035	12	16	30
Ferraz et al., 1991 [15]	The effectiveness of tipi in the treatment of hip and knee osteoarthritis-a preliminary-report	https://doi.org//10.1590/S0074-02761991000600054	4	5	11

WoS-CC: Web of Science Core Collection.

A total of 284 authors were identified, organized into 41 clusters, contributing to at least 1 article (Figure 1). Fiorentino, S. and Cáceres, A. were the authors who published the most (*n* = 6 and *n* = 5 papers per author, respectively), followed by Hernandez, J.F., Maia, C.S.F., and Urueña, C. (*n* = 3 papers per author). In relation to citations, Cavallaro, L., Ruffa, M.J., and Wagner, M.L. (*n* = 159 per author), Cáceres, A. (*n* = 151), and Calagno, M.L., Campos, R., and Ferraro, G. (*n* = 149 per author) were those with the most citations. 

The 55 selected articles were published in 37 different journals (Table 2), and only 7 of them were published in 2 or more. The Journal of Ethnopharmacology (JEP; JCR impact factor: 5.4), established in 1979 as the official journal of the International Society for Ethnopharmacology (ISE), contained 13 of the publications, including 6 of the 10 most-cited articles and 50.99% of the total citations (Table 2).

**Figure 2:**
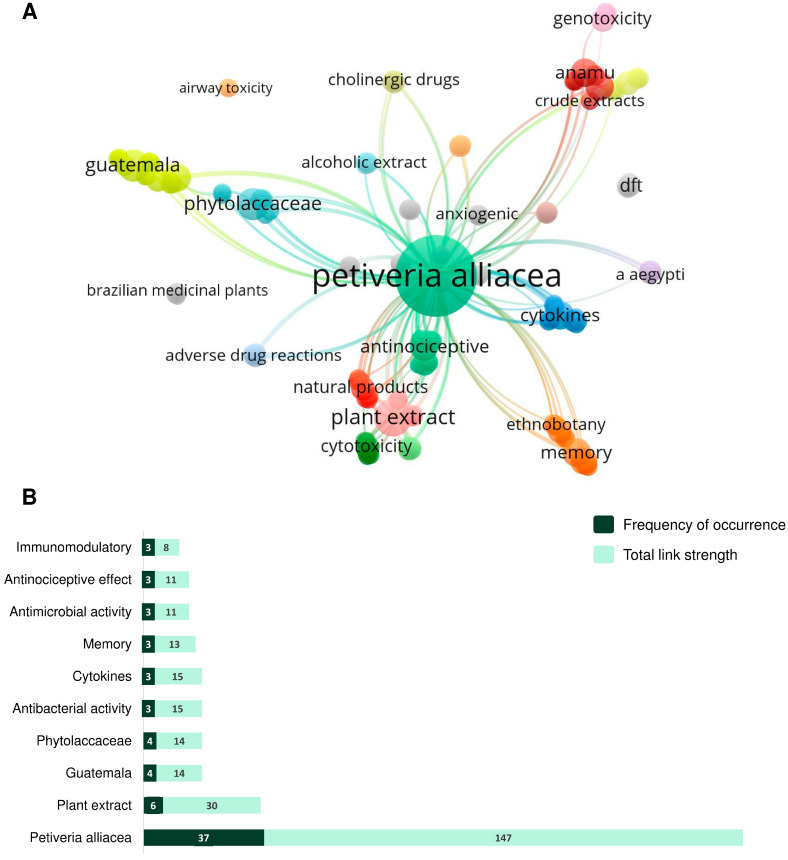
Network visualization of the co-occurrence of the keywords used by the authors of the selected studies using the VOS viewer software. Clusters are highlighted by different colors. The node size represents the frequency of the keyword, and the lines reveal the connections between the keywords (**A**). The top 10 most frequent words are represented in panel (**B**), associated with their total link strength.

Keywords represent essential topics or concepts covered in a research article. In this study, a total of 195 keywords were found, grouped into 28 interconnected clusters, as shown in Figure 2A. The top 10 most frequent keywords were: *Petiveria alliacea* (*n* = 37), plant extract (*n* = 6), Guatemala (*n* = 4), Phytolaccaceae (*n* = 4), antibacterial activity (*n* = 3), cytokines (*n* = 3), memory (*n* = 3), antimicrobial activity (*n* = 3), antinociceptive effect (*n* = 3), and immunomodulatory (*n* = 3) (Figure 2B).

**Figure 3:**
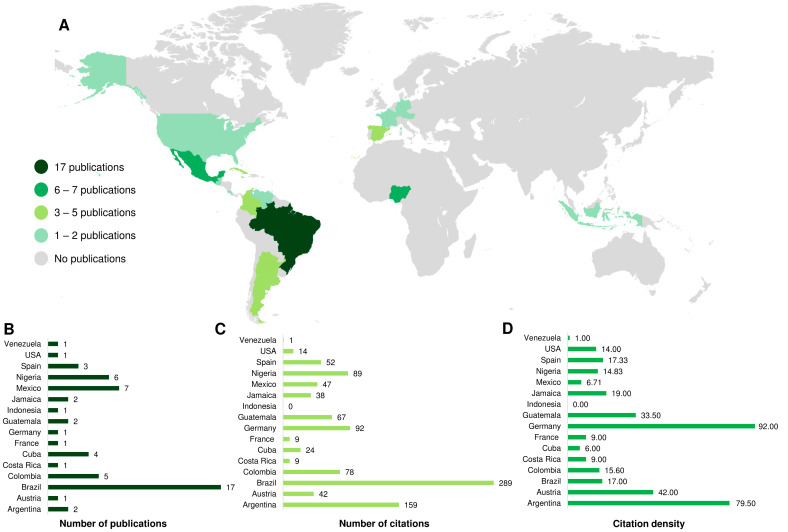
Worldwide distribution of selected articles (**A**) with the representation of countries from published articles (**B**), total number of citations (**C**), and citation density (**D**).

The scientific production related to *P. alliacea* properties is primarily distributed among a few countries (Figure 3). Brazil is the country with the highest number of publications (*n* = 17), followed by Mexico (*n* = 7) and Nigeria (*n* = 6). Colombia reached five publications, while Cuba and Spain presented four and three publications, respectively. Argentina, Guatemala, and Jamaica presented two publications each, while Venezuela, the United States, Indonesia, Germany, France, and Austria contributed one article each (Figure 3A,B). In terms of absolute number of citations, Brazil also ranks first (*n* = 289), followed by Argentina (*n* = 159) and Germany (*n* = 92) (Figure 3C). When evaluating the citation density, however, Germany takes first place (d = 92 per paper), followed by Argentina (d = 79.50 per paper) and Austria (d = 42 per paper).

### 2.2. Content Analysis

**Figure 4:**
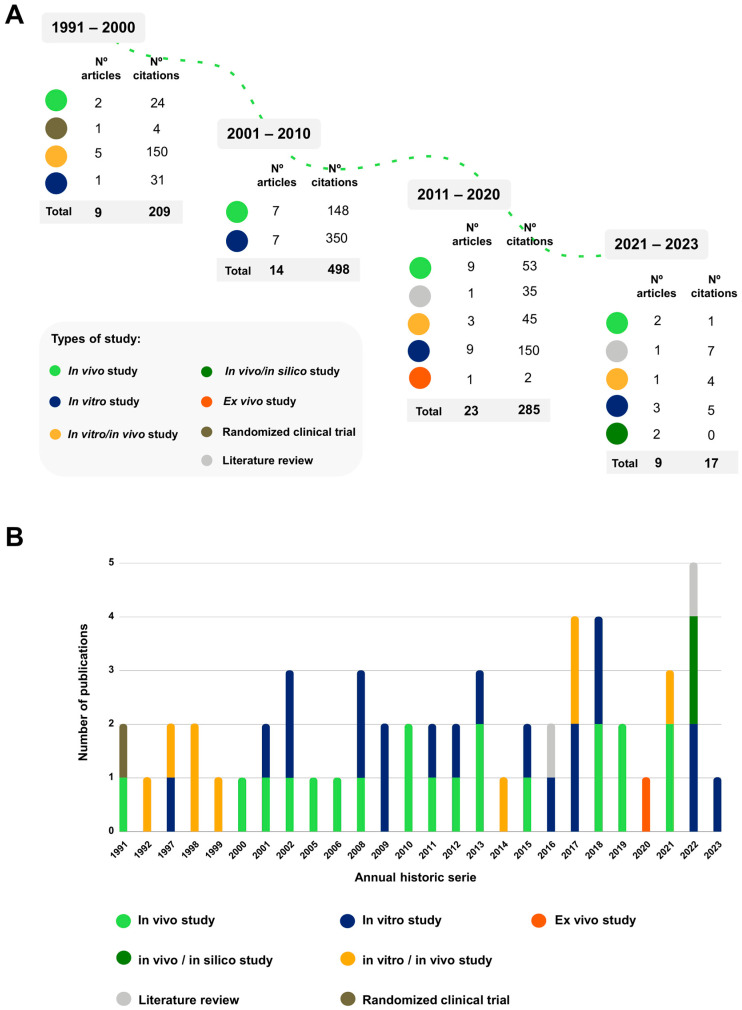
Historical series of publications by decade (**A**) and study types by year (**B**).

Based on publication frequency, the period ranging from 2011 to 2020 (*n* = 23) showed the highest number of published articles, which received an average of 1.86 citations per year. However, the highest number of citations occurred from 2001 to 2010 (*n* = 498), despite having fewer publications (*n* = 14), also presenting the highest average of annual citations per paper (2.05) (Figure 4). 

Regarding the methodological approach, 29 studies used in vitro assays, and in 20, it was the only approach adopted. In vivo assays, in turn, were present in 31 articles, being exclusive in 20. Studies based exclusively on in vitro assays have the highest number of citations (*n* = 536), also showing the highest citation density (26.85 citations per article) (Figure 4).

For the preparation of the extracts, mainly the leaves and roots of *P. alliacea* were used, highlighting the techniques of maceration, percolation, and Soxhlet extraction. The most frequently used solvents were ethanol and methanol in extraction and fractionation processes, including ethyl acetate. These and other relevant information are summarized in Table 3.

## 3. Discussion

In plants lies the main source of inputs for health care, whether in the industrial-technological development of medicines or in the popular use of their teas, ointments, and baths, among others. In this perspective, scientific research is essential for the discovery and characterization of new species with therapeutic and economic potential, as well as for subsidizing the safe and rational use of these species as a complementary healthcare strategy [59]. Understanding the beneficial and harmful effects of plant species, as well as the dose range and use profile associated with these effects, is valuable information, providing safer and/or more effective alternatives for the treatment of diseases.

It is noticed, however, that the study of medicinal plants often presents a slow and fragmented advance, rarely resulting in robust clinical studies or innovative products. Along with this, it is noted that its application as a complementary strategy for health care lacks foundation and standardization [59]. *P. alliacea*, in turn, is a species widely known by the population of the Americas and regions of Africa, having a diverse range of popular indications, some of which have already been the subject of investigation. To the best of our knowledge, this is the first scientific report with a bibliometric approach plus critical content analysis addressed to an Amazonian medicinal plant. Here, we focused on analyzing the scientific production of this medicinal herb, aiming to highlight its breadth, scope, impact, and stage of development, providing opportunities to identify the main active research groups and any gaps in the existing scientific literature.

Bibliometric studies offer a valuable approach to visualizing scientific production and analyzing metrics and trends within a specific field [60]. Conducting a bibliometric review requires a well-defined strategy to rescue studies related to the previously determined object of study. This strategy must be formulated considering Zipf’s law, a fundamental concept of bibliometrics, which associates the frequency of specific words (keywords) with terms that determine the essence of a scientific article [60,61,62]. In our study, using this approach, we found that the top 10 most frequent keywords aligned with our search strategy, which emphasizes the importance of thorough research and rigorous keyword choice.

Another analysis that this type of study allows us, through Lotka’s law, is to observe which are the most prolific authors on the subject analyzed and with which researchers they communicate [60,63]. In another way, Lotka’s law seeks to understand the trends on the subject and the vision of the connections between research networks [64]. This observation is important to know the worldwide contribution of researchers on a theme. We identified 285 researchers involved in the study of *P. alliacea*, dedicated to elucidating its toxicological and pharmacological properties. Despite the interest of several research groups of different nationalities, the connection between them is scarce, one of the factors that can contribute to the overlapping of trials and difficulty in advancing to new levels of research. The formation of collaboration networks, despite the contiguity of articles and harmony of results, is practically non-existent.

The third law of bibliometric study is Bradford’s law, which turns to the evaluation of the journals where the selected studies were published [60,65,66,67]. In this analysis, we used quantitative parameters, such as the Journal Citation Reports (JCR), the number of articles published, the number of citations received, or even the density of citations, to measure the participation and impact of journals in the dissemination of the knowledge investigated [65]. In our prospection, the journals had an average impact factor of 2.14 ± 2.15 (0–7.9), with 59% of the journals having JCR ≥ 2.0 and 31.5% > 5.0. JEP, which in 2023 presents JCR = 5.4, stood out for the number of articles on the biological effects of *P. alliacea*, having published 13 studies, while other journals did not exceed 2 articles. Further, considering that the articles published in JEP had 509 citations, equivalent to 51.16% of the total number of citations obtained by the selected articles, we can point to the journal of the International Society for Ethnopharmacology as the most influential journal in disseminating knowledge about *P. alliacea* bioactive properties.

Because it is a widely distributed species in the Americas, especially in the Amazon region, but also in Mexico and the Caribbean, its popular use is widespread in traditional communities of the region countries [27]. In relation to scientific research on its bioactive properties, we found that it is concentrated in the Americas (*n* = 41), mainly South America (*n* = 25), with emphasis on Brazil, the country that hosted 17 of the published studies. Central and North America hosted eight published studies each, with emphasis on Mexico, home to seven studies. Interestingly, however, studies originating in Africa (*n* = 6), Europe (*n* = 6), and Asia (*n* = 1) were also located, showing that the interest in its potential goes beyond the borders of its cultural insertion. Brazil’s leadership in the subject is confirmed by the impact of published studies, which have up to 289 citations. Moreover, four of these studies are among the ten most cited. This position may be related to the strong presence of groups dedicated to the study of medicinal plants in the country, greatly stimulated by the local biodiversity [68]. Despite this, we noticed that the studies were developed independently by specific institutions, evidencing the lack of interaction between research groups and the formation of networks, as previously discussed.

After the metric design of the dissemination and scientific impact of the *P. alliacea* knowledge, we turn to the paper’s content to expose the main interests in relation to the bioactive properties of the plant, the derivatives and formulations explored, the methodological strategies applied, and conclusions achieved, which are summarized in Table 2. This approach allows us to map the state-of-the-art study and expose its limitations, gaps, and perspectives.

Our research identified the first published studies related to the subject, the article “The effectiveness of tipi in the treatment of hip and knee osteoarthritis—A preliminary report” by Ferraz et al. [15] and “Evaluation of antinociceptive effect of *Petiveria alliacea* (guiné) in animals” by de Lima et al. [16], both published in Supplementary Volume 2 of the 86th edition of the Brazilian journal ‘Memórias do Instituto Oswaldo Cruz’, in 1991. Interestingly, the first study tracked on the species was a randomized clinical trial that explored a *P. alliacea* infusion as a therapeutic alternative for osteoarthritis treatment, which promoted superior pain relief compared to placebo despite having a reduced sample. Even more impressive is that, after 32 years, no further clinical trials related to the species have been identified. This occurrence could be initially related to the scarcity of studies that could subsidize clinical studies, a very present reality when it comes to the evaluation of medicinal plants. As previously mentioned, the lack of continuity and fragmentation of studies constitute a significant barrier to the advancement of herbal medicine research and development [69]. 

The second study tracked also addresses the antinociceptive property of the species, but this time in murine models, confirming its analgesic potential in inflammatory contexts. Since then, the in vivo study, using animal models, has been the most applied, being present in 55.6% of the articles [2,6,9,21,22,23,24,25,26,28,30,34,36,37,41,42,44,45,48,50,53,54,55,56,57,58]. This is followed by in vitro tests, present in 27 works [2,6,9,18,21,22,23,24,25,26,28,30,34,36,37,41,42,44,45,48,50,53,54,55,56,57,58]. 

In fact, between 2001 and 2010, the use of in vitro and in vivo techniques resulted in the production of 14 articles [18,39,40,41,42,43,44,45,46,47,48,49,50,51]. In this decade, however, the highest number (*n* = 494) and density of citations (d = 35,3 per paper) stood out. In the following decade, with the publication of 23 papers [10,11,13,14,20,21,22,23,24,25,26,27,28,29,30,31,32,33,34,35,36,37,38] and a greater diversity of methodological approaches, including a literature review and ex vivo methods, we verified a significantly smaller impact, reaching just over half of the citations (*n* = 278) of the previous period and having a citation density (d = 12 per paper) three times lower.

It is also interesting to note the progression in the number of publications over the years, which was 0.9 articles/year in the first decade, increasing in the following decades to 1.4 and 2.3 articles/year. Between 2021 and 2022, eight articles were published [2,4,6,7,8,9,12,19]. This, however, points to the continuity of the progression, reaching four articles/year. In this last period, we also observed the inclusion of computational tools (in silico methods) to investigate the properties and mechanisms of *P. alliacea* [8].

Regarding the plant derivatives used in the studies, leaves [2,4,5,6,8,9,10,11,14,15,18,19,20,21,22,23,24,25,26,29,30,32,34,35,36,38,41,42,44,45,46,48,50,56,57], roots [7,16,33,37,39,43,47,49,51,52,53,55], and stems [16,21,24,26,36,37,39,45,49,51,58], respectively, were the most used for the production of extracts and fractions. Maceration [2,4,6,7,9,18,19,23,25,29,35,37,41,43,45,47,48,50,51,52,53,54,55] and Soxhlet [14,20,26,36,42] were the most used techniques, and ethanol [2,4,6,8,9,14,19,20,25,28,29,35,36,40,41,42,43,44,45,47,48,51,52,53,54,55], methanol [2,8,10,14,18,23,24,25,37,48,50,51,55], and hexane [2,10,19,41,42,43,50,51,55,58] were the main solvents. Additionally, we found that only 16 of the 54 selected articles performed phytochemical prospecting. Among the identified compounds are benzaldehyde [24,29,30,44], dibenzyl disulfide [10,25,30,31], petiveral [25,31,45], and myricetin [10,25] as the most cited.

We verified a great diversity of activities being approached, aligned with the range of *P. alliacea’s* traditional indications, whose first formal records date back to the colonial period, when it was used by enslaved people, both for its therapeutic benefits and to induce intoxication [25,27]. Doses between 10 [8] and 5000 mg/kg [35] have been tested in the investigation of about 20 different biological activities in vivo, with its toxicity (*n* = 9), immunomodulatory (*n* = 7), antimicrobial (*n* = 7), antinociceptive (*n* = 5), anti-inflammatory (*n* = 5), antineoplasic (*n* = 5), and antioxidant (*n* = 4) properties being the most frequent. The in vitro concentrations tested range from 5 to 1000 µg/mL [6,10,18,20,33,39,46,58].

Toxicity studies investigated the cytotoxicity [6], genotoxicity and acute [58], and sub-chronic [46] toxicity of *P. alliacea*. In general, the tried extracts and fractions showed low toxicity, except for the described toxicity against cancer cells [48], based on their potential anticancer property. In addition to the most investigated actions, its behavioral effects have also been demonstrated, reducing depressive and anxious-like behaviors, antiparasitic, antifungal, anticoagulant, anticholinesterase, and insecticide activity, among others. These findings point to a multi-target pattern of action, with the potential to treat diseases and comorbidities simultaneously, which, when used rationally, can promote significant gains in terms of therapy and quality of life for patients [3].

Interestingly, despite the pre-clinical and clinical approaches that confirm its properties, no medicine, not even an herbal medicine, has been developed from this species or its metabolites. Unfortunately, this is an overview of studies involving medicinal plants, especially in South American countries, which, in the case of *P. alliacea*, concentrates most of the studies on their biological activities. 

We observed that none of the studies carried out with the plant were continued, in the sense of starting, for example, the toxicity study and carrying out all the steps for the characterization of its toxicological safety. In many cases, a research group performs only the acute oral toxicity, but not the subchronic or chronic, or even only evaluates the basic parameters. A similar occurrence happens in pharmacological evaluation. For example, Gomes et al. [47] performed the antinociceptive evaluation of fractions of *P. alliacea* extracts. However, they did not report the toxicity data for the fractions. Thus, they discuss the effects on nociception observed based on phytocompounds described in the literature from other derivatives of the species. 

It is known that metabolite plant content can be altered by numerous factors, such as the part used, seasonality, extraction type, comminution degree of the particles submitted to extraction, etc. Therefore, it is essential that the assessment of pharmacological properties is always accompanied by the phytochemical profile of the natural product being studied. A chemical content investigation would not only help to validate the results but could also direct research on possible compounds involved, especially in the current scenario, in which in silico studies for the characterization of the structure and activity of compounds or their improvement may be realized. On the other hand, the effect of the medicinal species is not always mediated by a single compound, but by a set of them, called a phytocomplex [70]. Such characteristics do not exclude its therapeutic potential; however, it demands a rigorous toxicological characterization, as well as the standardization of the preparation to be tested. 

This lack of dialogue between chemical and biological analyses is largely due to the fragmentation of studies by different research groups and the lack of an effective network for investigating the biological activity of plants with the aim of generating a product, following all the necessary steps, especially in countries in South America. Such a scenario is in contrast with reality, where one of the largest and richest biomes in the world is located on this continent, whose pharmacological potential still needs to be investigated [71]. Thus, this work can contribute to reinforcing the necessity of continuity of research carried out with *P. alliacea*, pointing out study trends through the presented metrics and reinforcing the gaps in the existing knowledge.

## 4. Materials and Methods

We performed a bibliometric-type analysis, as previously reported by our research group [72].

### 4.1. Data Source and Collection

**Figure 5:**
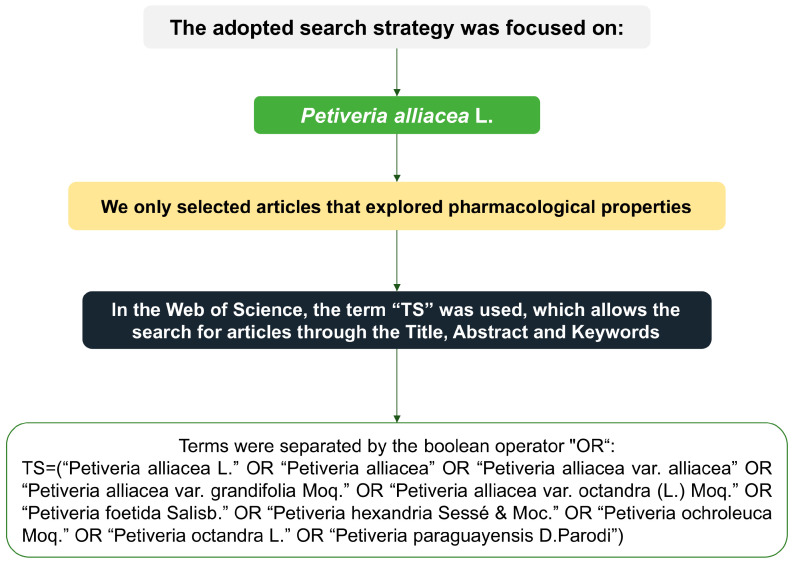
Schematic representation of the search strategy applied to retrieve selected articles.

The Web of Science Core Collection (WoS-CC) database was used to perform a global search on *P. alliacea.* The data collection was performed on 3 August 2023; the applied search strategy is described in Figure 5.

### 4.2. Establishment of Inclusion Criteria

Selected documents were original articles or review articles that explored the pharmacological and toxicological properties of *P. alliacea*, and no language restrictions were applied. Conference papers, editorials, letters to authors, proceedings papers, articles not freely available, studies focused on ethnopharmacology, and publications that did not assess the pharmacological properties of *P. alliacea* for therapeutic purposes were excluded.

### 4.3. Articles Selection

After defining the inclusion criteria, two independent researchers searched for articles through the WoS-CC platform. In cases of divergence about the inclusion of articles, a third experienced researcher was consulted. Subsequently, a survey of citations of selected articles was performed in other databases (Scopus and Scholar Google).

### 4.4. Bibliometric Analysis

Based on the data obtained from WoS-CC, bibliometric analyses were performed. Here, we collected information about articles’ titles, author’s name, number of citations, citation density by country, journal’s name, journal’s impact factor (considering JCR 2022, ©2023 Clarivate), author’s keywords, countries, and institutions. The VOSviewer software (version 1.6.16) was used to visualize the metrics listed [72]. The generated networks are represented by nodes (clusters) and lines (connections); the cluster size is directly proportional to the number of publications/citations of authors, frequency of occurrence of keywords, and institutions; the lines between each cluster represent the co-authorship network, the connection between keywords, or inter-institutional connection. Based on this, we also performed the total link strength between authors and keywords. To illustrate the global distribution of selected publications, the MapChart tool (https://mapchart.net/, accessed on 3 August 2023) was used.

### 4.5. Content Analysis

**Figure 6:**
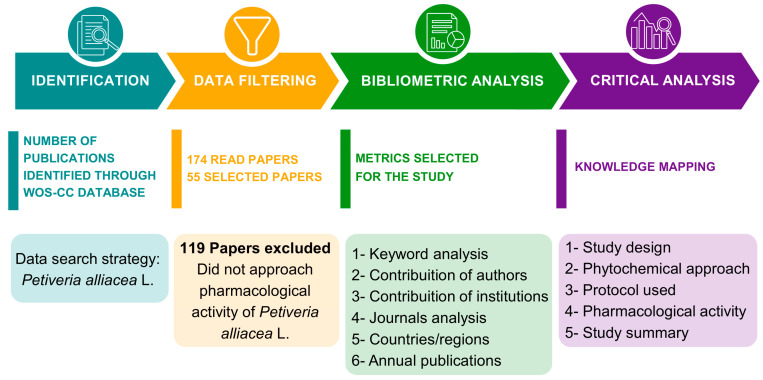
Methodological strategy applied to perform the critical analysis.

Considering the scarce scientific production on medicinal plants, mainly *P. alliacea*, it was decided to critically analyze the study designs, phytochemical approaches, protocol used, pharmacological activity, and study summary of the selected articles. With this information gathered, this review provides robust research on the use of *P. alliacea* for pharmacological purposes. Figure 6 demonstrates the methodological strategy applied to perform the critical analysis.

## 5. Conclusions

In this bibliometric analysis, we evidenced the paradox between the growing interest in investigating plants with therapeutic potential and the limitations and gaps in scientific research on the subject. Studies on this species, widely disseminated in the medicinal culture of American populations, especially the Amazon, demonstrated an important analgesic – Including through clinical trial – immunomodulatory, anti-inflammatory and antioxidant potential, among others. It is therefore necessary to deepen and advance scientific research, which can be enhanced by greater interaction between research groups. In this sense, this review presents a comprehensive overview of the research already carried out on the toxicity and pharmacological properties of *P. alliaceae*, its ecosystem of publications and researchers dedicated to the topic, in an intuitive and pragmatic way, providing insights for this subject advancement.

## Figures and Tables

**Table 2:** Journals that published studies involving *Petiveria alliacea*.

Journals	IF ^a^	Number of Articles	Number of Citations	Citation (%) ^b^
African Journal of Biotechnology	*	1	8	0.79
American Journal of Chinese Medicine	5.7	1	11	1.09
Biological & Pharmaceutical Bulletin	2	1	29	2.87
Bmc Complementary and Alternative Medicine	*	1	39	3.86
Boletin Latinoamericano y del Caribe de Plantas Medicinales y Aromaticas	0.7	2	4	0.40
Brazilian Journal of Pharmaceutical Sciences	1.3	1	8	0.79
Brazilian Journal of Pharmacognosy	1.6	2	14	1.39
Chemistry Africa	2.6	1	4	0.40
Chemotherapy	3.3	1	10	0.99
Chinese Journal of Integrative Medicine	2.9	1	2	0.20
Evidence-Based Complementary and Alternative Medicine	*	1	2	0.20
Farmaco	*	1	11	1.09
Fitoterapia	3.4	1	19	1.88
Immunopharmacology and Immunotoxicology	3.3	2	25	2.48
International Journal of Morphology	0.5	2	5	0.50
International Journal of Tropical Insect Science	1.2	1	1	0.10
Journal of Essential Oil Research	3	1	9	0.89
Journal of Ethnopharmacology	5.4	13	515	50.99
Journal of Medicinal Plants Research	*	1	12	1.19
Journal of Microbiology and Biotechnology	2.8	1	10	0.99
Latin American Journal of Pharmacy	0.2	1	3	0.30
Memorias do Instituto Oswaldo Cruz	2.8	2	16	1.58
Mutation Research	*	1	14	1.39
Pharmaceuticals	4.6	1	0	0.00
Pharmaceutics	5.4	1	0	0.00
Pharmacognosy Magazine	0.7	1	20	1.98
Phytochemistry	3.8	1	50	4.95
Phytomedicine	7.9	1	32	3.17
Phytotherapy Research	7.2	2	50	4,95
Plants-Basel	4.5	1	0	0.00
Preparative Biochemistry & Biotechnology	2.9	1	63	6.24
Revista de Biologia Tropical	0.6	1	9	0.89
Revista Virtual de Quimica	0.5	1	1	0.10
Spanish Journal of Agricultural Research	0.9	1	1	0.10
Veterinary Parasitology	2.6	1	7	0.69
Veterinary World	1.6	1	0	0.00
West Indian Medical Journal	0.1	1	6	0.59

^a^ IF: Impact Factor; ^b^ citation percentage considering the citation number of each journal and the total number of citations; * Journals without IF.

**Table 3:** Synthesis of selected articles on *Petiveria alliacea* L. in WoS-CC, highlighting phytochemical and pharmacological aspects and main findings.

Authors/Year	Design	Phytochemical Approach			Protocol Used		Pharmacological Activity	Study Summary
		Plant Parts Used	Type of Extraction	Phytochemical Constitution	Dose/ Concentration	Route and Period of Administration		
Clinical study								
Ferraz et al., 1991 [15]	Randomized clinical trial	Leaves and stems	Infusion method; Solvent: water	Not investigated	200 mL of 15 mg/mL solution	Oral administration; 3 times a day, for 3 weeks	Antinociceptive study	In this study, the authors investigated the antinociceptive potential of *P. alliacea* in 14 patients with osteoarthritis. The authors reported no statistically significant evidence that *P. alliacea* was superior to placebo in reducing the severity of pain. However, the authors emphasized the possibility that the small sample of patients compromised the statistical analysis performed.
In vivo study								
de Lima et al., 1991 [16]	Pre-clinical in vivo study (male and female mice)	Roots	Infusion method; Solvent: water	Not investigated	125–2000 mg/kg	Oral administration (gavage) or parenteral administration (intraperitoneal); Single dose	Antinociceptive study	In this study, the authors evaluated the effects of aqueous crude extract of *P. alliacea* on sedative and analgesic properties in mice and rats. The authors stated that the antinociceptive effect in acetic acid, acetylcholine, and hypertonic saline induced animal constrictions but not in hot-plate and tail flick tests. The authors also reported that *P. alliacea* did not produce any CNS depressor effect.
Queiroz et al., 2000 [52]	Pre-clinical in vivo study (male mice)	Roots	Maceration method; Solvents: ethanol and water	Not investigated	1000 mg/kg	Oral administration (gavage); Daily for 5 days, once a day	Immunomodulatory study	In this study, the authors investigated the effects of *P. alliacea* extract on the production of Th1-type and Th2-type cytokines and on NK cell activity in normal and infected mice. The authors suggest that *P. alliacea* administration up-regulates antibacterial immune response by enhancing both Th1 function and the activity of NK cells.
Morales et al., 2001 [51]	Pre-clinical in vivo study (male and female mice)	Roots	Maceration and percolation method; Solvents: hexane and chloroform, methanol and ethanol, water	Not investigated	1.25 g/kg	Parenteral administration (Intraperitoneal); Single dose	Neuropharmacological study	In this study, the authors screened four different South American plants to investigate neuropharmacological activities through the Irwin test method. Regarding *P. alliacea*, the authors stated that the extract from roots only lightly decreased spontaneous motor activity, and the leaf extract showed hyperexcitability. The authors emphasize that *P. alliacea* has little effect on behavioral, neurologic, and autonomic signs compared to other plants studied.
Lopes-Martins et al., 2002 [49]	Pre-clinical in vivo study (male rats)	Roots	Percolation method; Solvents: ethanol (66.7%)	Not investigated	31.4 and 43.9 mg/kg	Oral administration (gavage), single dose	Anti-inflammatory and analgesic study	In this study, the authors investigated the anti-inflammatory properties of *P. alliacea* crude extract administered to rats with pleurisy. The authors reported that the oral administration did not reduce the total number of leukocytes at the doses tested. However, the highest dose (43.9 mg/kg) reduced mononuclear cell migration, in addition to having an analgesic effect. The authors emphasized that the results provide a basis for folk medicine use, but further studies are necessary to elucidate the mechanism of anti-inflammatory and analgesic actions.
Gomes et al., 2005 [47]	Pre-clinical in vivo study (female mice)	Roots	Maceration method; Solvents: ethanol and water; Fractionation process with hexane and ethyl acetate	Not investigated	100 and 200 mg/kg	Parenteral administration (Intraperitoneal), single dose	Antinociceptive study	In this study, the authors evaluated four fractions (acetate, hexanic, hydroalcoholic, and precipitated hydroalcoholic) from *P. alliacea* roots for antinociceptive effects. The authors also evaluated psychomotor and myorelaxant activities. It was reported that all fractions showed reduced locomotor activity on the open field test; however, different fractions had different antinociceptive potentials.
García-Gonzáles et al., 2006 [46]	Pre-clinical in vivo study (male mice)	Leaves	Infusion method; Solvents: water	Not investigated	1000 and 2000 mg/kg (both acute toxicity and sub-chronic toxicity)	Oral administration (gavage); 5 consecutive days for 3 weeks (acute toxicity) and 5 consecutive days for 8 weeks	Toxicological study (acute and sub-chronic)	In this study, the authors tested the effects of an aqueous extract of *P. alliacea* on acute and sub-chronic toxicity in male mice. The authors reported that no mortality nor any toxicity signs could be observed. The authors also stated that no significant differences in intestinal motility or blood glucose levels could be found.
Gomes et al., 2008 [43]	Pre-clinical in vivo study (female mice)	Roots	Maceration method; Solvents: ethanol and water; Fractionation process with hexane and ethyl acetate	Not investigated	100 and 200 mg/kg	Parenteral administration (Intraperitoneal) and oral administration (gavage); Single dose for each route of administration	Neuropharmacological study	In this study, the authors investigated the neuropharmacological properties of fractions from *P. alliacea* hydroalcoholic extract. The authors reported that all fractions suggested central depressant activity on the open field test and indicated an absence of anxiolytic-like effect on the elevated plus-maze test. The authors conclude that *P. alliacea* contains biological substances that have significant depressant and anticonvulsant potentials, supporting folk medicine use.
Blainski et al., 2010 [40]	Pre-clinical in vivo study (male mice)	Whole plant, aerial parts, and roots	Turbo-extraction method; Solvent: ethanol (50%)	Not investigated (only total flavonoid content)	300, 600, and 900 mg/kg (All extracts)	Oral administration (gavage); Single dose	Behavioral study-anxiolytic activity	In this study, the authors investigated the behavioral effects of three different types of extracts from *P. alliacea* in mice using the open field and elevated plus maze apparatus. The authors described that the whole plant extract caused anxiolytic-like effects, and the aerial parts extract induced anxiogenic-like effects. The authors also suggested that the total flavonoid content may have a pivotal role in the obtained results, but further study is required.
Maia et al., 2010 [39]	Pre-clinical in vivo study (female rats)	Roots	Hydroalcoholic extract (preparation not described)	Not investigated	18 mg/kg	Oral administration (gavage), single dose, 5th day of pregnancy	Toxicological study	In this study, the authors investigated the effect of a hydroalcoholic extract of *P. alliacea* roots on fetal and placental development in female rats. The extract caused a significant reduction in the number of implantation sites but no histological alterations in these sites and placenta. The authors also observed no significant alterations in the number, length, or weight of the offspring.
Duharte et al., 2011 [38]	Pre-clinical in vivo study (female mice)	Leaves and stems	There was no extraction process. The plant drug (*P. alliacea*) was dissolved in a solution of carboxymethylcellulose and water	Not investigated	400 and 1200 mg/kg	Oral administration (gavage); Daily administration for 8 days, once a day	Immunomodulatory study	In this study, the authors determined the protective properties of *P. alliacea* on 5-FU immunosuppressed animals. The authors found that the group treated with the highest dose of *P. alliacea* was less affected by 5-FU-induced immunosuppression compared with the other treated groups. These results suggest that the plant could be used in patients under antineoplastic regimens to avoid the deleterious adverse effects of immunosuppressive drugs.
de Andrade et al., 2012 [35]	Pre-clinical in vivo study (female and male rats)	Leaves, roots, and stems	Maceration method; Solvent: ethanol (70%)	Not investigated	2000 mg/kg or 5000 mg/kg (Oral acute toxicity); 900 mg/kg (behavioral assays)	Oral administration (gavage) for both acute toxicity and behavioral assays; Single dose for both assays	Behavioral plus oxidative study	In this study, the authors evaluated the behavioral and oxidative stress effects of hydroalcoholic extracts of *P. alliacea* in rats. The authors reported increased locomotor activity, as well as antidepressant and anxiolytic, in behavioral tests. The oxidative stress assessment showed pro-oxidative effects in vivo and in vitro. The authors also suggest that the polyphenols found in *P. alliacea* may have neuronal mechanisms that potentially regulate anxiety as well as oxidative stress.
Fletes-Arjona et al., 2013 [33]	Pre-clinical in vivo study (male rats)	Roots	There was no extraction process. The roots were used fresh	Not investigated	150 mg	Inhalation route; Single exposure for 3 min	Toxicological study (acute)	In this study, the authors evaluated the morphologic alterations in the respiratory tract of female rats induced by the inhalation of *P. alliacea* steams. The authors reported that the main histological and morphological alterations were hyperplasia on the trachea epithelium, signs of elevated secretion from goblet cells, and vascular congestion on the bronchiole. The authors also suggest that the morphologic alterations happened due to coumarins present in the chemical composition of *P. alliacea.*
Christie and Levy, 2013 [34]	Pre-clinical in vivo study (male rats)	Leaves	Decoction method; Solvent: water	Not investigated	200 and 400 mg/kg	Oral administration (gavage); Single dose	Antidiabetic study	In this study, the authors evaluated the hypoglycemic effect of *P. alliacea* on a model of diabetes induced with streptozotocin, as well as the effect of *P. alliacea* in normoglycemic female rats. The authors report that they did not observe a hypoglycemic effect in the model used. The authors state that the results are contrary to the medicinal use of the species, which does not support the use of *P. alliacea* for hypoglycemic purposes.
Silva et al., 2015 [29]	Pre-clinical in vivo study (female and male rats)	Leaves	Maceration method; Solvent: ethanol (70%)	Organosulfur compounds	900 mg/kg	Oral administration (gavage); Single dose	Behavioral study—procognitive activity	In this study, the authors investigated the activities of *P. alliacea* on cognition (learning and memory). The authors reported that there were effects on the memory process and improved learning in the tests performed in the study. They also emphasized the importance of studies that investigate the pharmacological mechanisms involved in these observed activities.
Gutierrez and Flores, 2018 [14]	Pre-clinical in vivo study (male mice)	Leaves	Soxhlet extraction (SE); Solvent: methanol	Not investigated	100, 200, and 400 mg/kg	Oral administration (gavage); Daily administration for 6 days, once a day	Anti-inflammatory and immunomodulatory study	In this study, the authors proposed to investigate the effect of *P. alliaceae* in a murine model of chronic asthma to assess the impacts on airway inflammation. The authors report that *P. alliaceae* can inhibit airway inflammation and regulate chemokines and cytokines, improving lung conditions in the asthma model used.
Garcia-Perez et al., 2018 [20]	Pre-clinical in vivo study (female and male rats)	Leaves and stems	Soxhlet extraction (SE); Solvent: ethanol (96%). Fractionation process with hexane, ethyl acetate, and water	Hexanic fraction: Phytol, (R)-(-)-(Z)-14-Methyl-8-hexadecen-1-ol, 3-Eicosyne, (3,7,11,15-Tetramethyl-2-hexadecen-1-ol), 1R,2c,3t,4t-Tetramethyl-cyclohexaxe; Ethyl acetate fraction: Phytol, (R)-(-)-(Z)-14-Methyl-8-hexadecen-1-ol, 3-Eicosyne, (3,7,11,15-Tetramethyl-2-hexadecen-1-ol), 2-Fluorobenzoic acid, 2-formyl-4,6- dichlorophenyl ester; Aqueous fraction: Methyl β-dimethylaminoisobutyrate, 1-(2-Hydrohyethyl)-1,2,4-triazole, 2-butanone,4-(dimethylamino)-3-methyl, Cis-3-Hydroxy-2-propylpiperidine	For the acute toxicity test, *P. alliacea* powder was administered at 2000 mg/kg. For the repeated oral toxicity test, the dose of 1000 mg/kg was chosen	Oral administration (gavage); Acute toxicity: single dose; Repeated toxicity: daily administration for 28 days, once a day	Toxicological study (acute and subchronic)	In this study, the authors investigated the acute and repeated toxicity of a powdered suspension of leaves and stems of *P. alliacea*. The authors reported that there was no death of the animals and no adverse effects that impacted the weight, general condition, and histopathological characteristics of the animals were observed.
Alves et al., 2019 [13]	Pre-clinical in vivo study (male rats)	Whole plant	No extraction method was adopted. *Petiveria alliacea* powder (vegetable drug) was used	For the phytochemical characterization, the authors used fresh leaves of *P. alliacea*, in which the following were identified: Dimethylsulfide, Diethylsulfide, α-Pinene, ß-Pinene, Di-n-propylsulfide and Nerolidol	25 or 50 mg of the dried plant were burned to charcoal in a specific inhalation device	Inhalation route: Single exposure (each animal remained for 60 s in the inhalation chamber)	Behavioral study (anxiolytic activity) plus toxicological study (evaluation of lung inflammation)	In this study, the authors investigated the composition and exposure of animals to *P. alliacea* smoke. The objective of the study was to evaluate the potential anxiolytic and toxic effects of this exposure. The authors reported that there was no anxiolytic effect in the animals, but after histological analysis, there was possible pulmonary inflammation.
Caicedo-Pinto et al., 2019 [11]	Pre-clinical in vivo study (male rats)	Leaves	Infusion method; Solvent: water	Not investigated	The average consumption of *P. alliacea* infusion per animal was 7.59 mL	Oral administration (via drinking water); Administration period not specified	Behavioral study—anxiolytic activity	In this study, the authors evaluated the potential effect of *P. alliacea* and cholinergic drugs on cognitive behavior. The authors described that *P. alliacea* had an anxiolytic and antidepressant effect, and when combined with nicotine, it demonstrated potentiation of the anxiogenic effect.
Oyeleke et al., 2021 [7]	Pre-clinical in vivo study (female chickens)	Roots and leaves	Maceration method; Solvent: water	Not investigated	15, 30, and 45 g/L (both root and leaf extract)	Oral administration (via drinking water); Twice per week for 10 weeks	Antimicrobial, immunomodulatory, and hepatomodulatory study	In this study, the authors investigated the antimicrobial, immunomodulatory, and hepatomodulatory activities of *P. alliacea*. Based on tests, the authors demonstrated that *P. alliacea* has the activities they set out to investigate.
Mustika et al., 2022 [4]	Pre-clinical in vivo study (male rats)	Leaves	Maceration method; Solvent: ethanol (70%)	Not investigated	50, 100, and 200 mg/kg	Oral administration; 14 days (once a day)	Anti-inflammatory study	In this study, the authors investigated the extract of *P. alliacea* using a self-nanoemulsifying drug release model in an animal model with insulin resistance. The study evaluates the levels of interleukin (IL)-6 and TNF-α with the aim of evaluating the effects of *P. alliacea* on inflammation associated with diabetes. The authors reported that the proposed model enabled the reduction in insulin resistance, as well as suppressed the levels of (IL)-6 and TNF-α.
In vitro study								
Villar et al., 1997 [56]	Pre-clinical in vitro study (human platelets)	Leaves	Decoction method; Solvents: water	Oxidable compounds	4 μL	Not applicable	Hematological study	In this study, the authors assayed 17 different aqueous plant extracts used in Guatemala for platelet antiaggregant activity. Regarding *P. alliacea*, the authors stated that the aqueous extract produced an inhibitory effect on the aggregation of washed human platelets induced by thrombin in a concentration-dependent form.
Benevides et al., 2001 [50]	Pre-clinical in vitro study (Phytochemical isolation)	Leaves, stems, and roots	Maceration method; Solvents: dichloromethane, methanol; Fractionation process with methanol, water, chloroform, ethyl acetate, and hexane	Dipropyl disulfide, Dibenzyl sulfide, Dibenzyl disulfide, Dibenzyl trisulfide, Dibenzyl tetrasulfide, enzyl hydroxymethyl sulfide, Di(benzyltrithio) methane	20, 10, 1, and 0.1 μg for pure compounds and 100, 50, 30, and 20 μg for the crude extracts or fractions, respectively	Not applicable	Antimicrobial and phytochemical study	In this study, the authors conducted a phytochemical investigation on the extract of *P. alliacea* roots, isolating five substances known as polysulfides. The authors also tested different fractions for antifungal activity. The authors reported that the fractionation led to the isolation of dipropyl disulfide, dibenzyl disulfide, dibenzyl trisulfide, benzylhydroxymethyl sulfide, and di(benzyltrithio) methane, the last three substances being new compounds.
Ruffa et al., 2002 [17]	Pre-clinical in vitro study (liver cells)	Leaves	Maceration method; Solvents: methanol	Not investigated	15.5–1000 μg/mL	Not applicable	Cytotoxicity study	In this study, the authors evaluated eight different methanolic extracts on cytotoxic effects on hepatocellular carcinoma cell lines. Regarding *P. alliacea*, the authors stated that the extract did not show any inhibitory effect on Hep G2 in the range of doses assayed (15.5–1000 μg/mL).
Ruffa et al., 2002 [48]	Pre-clinical in vitro study (bovine cells)	Leaves and stems	Maceration method; Solvents: methanol; Fractionation process with ether oil	Not investigated	10 mg/mL	Not applicable	Antiviral study	In this study, the authors assayed five different medicinal plants used in Argentina to detect the inhibition of viral growth. Regarding *P. alliacea*, the authors stated that the extract inhibited bovine viral diarrhea virus replication but showed no activity against herpes simplex virus type 1, poliovirus type 1, adenovirus serotype 7, and vesicular stomatitis virus type 1. The authors conclude that, among the five different plants studied, *P. alliacea* was worth studying in the future.
Adomi, 2008 [45]	Pre-clinical in vitro study (bacteria)	Leaves	Maceration method; Solvents: water and absolute ethanol	Thiobenzaldehyde s-oxide, senfol, coumarin, pinitol, dibenzyl sulfide, s-(2-hydroxyethy)phenylmethaenethiosulfinate), 3,5-diphenyltritiolan, dibenzyl trisulfide, leridol 5-methyl ether, 4-ethylpetiveral, glutamyl-S-benzyl cysteine, lignoceric acid, myricitrin	250–1000 mg/mL	Not applicable	Antibacterial study	In this study, the author screened three plants for antibacterial activity. Regarding *P. alliacea*, the author reported that both aqueous and ethanolic extracts did not show antibacterial activity.
Urueña et al., 2008 [44]	Pre-clinical in vitro study (tumoral cells)	Leaves and stems	Reflux method; Solvents: ethanol (96%); Fractionation process with ethyl acetate	Not investigated	125 μg/mL	Not applicable	Anticancer study	In this study, the authors assessed multiple in vitro biological activities of an ethyl acetate fraction of *P. alliacea* over tumoral cell lines. The authors reported that the fraction altered actin cytoskeleton organization, inducing G2 cell cycle arrest and causing apoptotic cell death. The authors also suggested that *P. alliacea* exerts multiple biological activities and has potential as an antitumor agent.
Schimdt et al., 2009 [42]	Pre-clinical in vitro study (T cell lineage and fibroblasts; bacteria)	Leaves, aerial parts, and flowers	Soxhlet and ultra-sound method; Solvents: n-hexane, ethanol	Not investigated	100 μg/mL	Not applicable	Immunodulatory and antimicrobial study	In this study, the authors investigated twelve plants used in Brazilian traditional medicine for various biological activities. Regarding *P. alliacea*, the authors reported that ethanolic extract showed promising wound-healing properties related to TNF-α suppression and Nf-κB binding.
Guedes et al., 2009 [41]	Pre-clinical in vitro study (microorganisms)	Aerial parts	Dynamic maceration method; Solvents: hexane, methylic alcohol, ethylic alcohol, ethanol (70%), purified water	Not investigated (total flavonoid and polyphenol)	240–3960 μg/mL	Not applicable	Antimicrobial study	In this study, the authors evaluated the antifungal and antibacterial activity of several crude leaf extracts of *P. alliacea* against different strains of bacteria and yeasts. The authors reported that promising results were shown for the 70% ethanolic extract, which presented a minimum inhibitory concentration from 250 to 760 μg/mL for yeast.
Adejumo et al., 2011 [37]	Pre-clinical in vitro study (sickle cell)	Roots	Maceration method; Solvent: methanol. Fractionation process with methanol-water mixture and chloroform	Alkaloids, saponins, and tannins	10, 1.0, and 0.1 mg/mL (both methanolic extract and aqueous fraction)	Not applicable	Hematological study	In this study, the authors evaluated the anticycling activities of aqueous fractions of *P. alliacea* on hematological cells. The authors reported that three fractions showed significant anticycling activity. The phytochemical screening revealed the presence of saponins, tannins, and alkaloids. The authors also emphasize that the popular use of *P. alliacea* for sickle cell disease is justified.
Santander et al., 2012 [36]	Pre-clinical in vitro study (monocytic cells)	Leaves and stems	Soxhlet extraction (SE); Solvent: ethanol (96%). Fractionation process with ethyl acetate (organic fraction) and water (aqueous fraction)	Not investigated	31.2, 62.5, 125, 250 and 500 μg/mL (both aqueous and organic fractions)	Not applicable	Immunomodulatory study	In this study, the authors evaluated the immunomodulatory activity of aqueous and organic plant fractions from *P. alliacea* using human monocyte-derived dendritic cells. The authors found that the aqueous fraction induced morphological changes in dendritic cells and pro-inflammatory cytokines secretion. The fraction also increased NF-kB gene expression while down-regulating TGF β gene expression. The authors also emphasize that the organic fraction by itself showed no immunomodulatory activity.
Pacheco et al., 2013 [34]	Pre-clinical in vitro study (microorganisms)	Leaves	In this study, soft extracts and blended extracts were prepared; Solvents: hydroalcoholic solution and isopropyl alcohol (soft extracts); hydroalcoholic solution (blended extracts)	Not investigated	10 μL of each extract	Not applicable	Antimicrobial study	In this study, the authors evaluated the antimicrobial activity of 13 different extracts from *P. alliacea*. The authors reported that nine extracts presented antibacterial activity, with blended extracts more potent than soft extracts. The authors also reported that B8 and E3, the most concentrated extracts, had the greatest spectrum of antibacterial action, being related to apolar substances.
Kerdudo et al., 2015 [30]	Pre-clinical in vitro study (microorganisms)	Aerial parts	Hydrodistillation method (Clevenger apparatus). Solvent: water	Benzaldehyde, toluenethiol, 1,2,5-trithiepane, dibenzyl disulfide, phytol, ethyl linolenate, dibenzyl trisulfide	0.5% and 0.05% *w*/*v* of essential oil	Not applicable	Antimicrobial study	In this study, the authors investigated the potential antimicrobial activity of *P. alliacea* from different regions of Martinique. The authors reported that the essential oil extracted from one of the selected sites inhibited the growth of several microorganisms, including some multiresistant strains. The authors also commented on the importance of studies with fractionated oil to identify the antimicrobial molecules involved in the observed activity.
Murray et al., 2016 [28]	Pre-clinical in vitro study (enzymatic assay)	Whole plant	Three types of extract were prepared: aqueous (whole plant, roots), 65% ethanolic (whole plant, leaves and trunk, roots), and 96.5% ethanolic (roots only). A tincture was also prepared using rum as solvent (alc/vol = 63%). In total, seven extracts were prepared; Aqueous type extracts were prepared by the infusion method; the ethanolic ones, by maceration	Not investigated	In this paper, the adopted concentrations were not clearly presented	Not applicable	Pharmacokinetic study	In this study, the authors carried out an analysis of the effects of dibenzyl trisulfide (DTS) on the activities of the cytochrome P450 enzyme, an important enzyme for the process of drug metabolism. The authors demonstrate in their study that DTS acts as a cyp450 inhibitor and consider *P. alliacea* a valuable species for many studies that seek to investigate the impact of high DTS content on drug interactions important for clinical therapy.
Gutierrez and Hoyo-Vadillo, 2017 [26]	Pre-clinical in vitro study (RAW 264.7 murine macrophage cells)	Leaves	Soxhlet extraction (SE); Solvent: ethanol	Not investigated	50, 100 and 200 μg/mL	Not applicable	Anti-inflammatory study	In this study, the authors evaluated *P. alliacea* in a murine model of RAW264 macrophages to analyze whether there is inflammation attenuation. The authors report that *P. alliacea* suppressed the secretion of prostaglandin and other inflammatory mediators. With the results obtained in this work, the authors state that *P. alliacea* has antioxidant activity and great anti-inflammatory potential.
Flota-Burgos et al., 2017 [23]	Pre-clinical in vitro study (nematode larvae)	Leaves and stems	Maceration method; Solvent: methanol	Not investigated	600, 300, 150, 75, 37.5, 18.7, and 9.3 μg/ml	Not applicable	Anthelmintic study	In this study, the authors evaluated the ability of *P. alliacea* to control nematodes. The authors reported that in their study, they observed a strong effect of preventing the hatching of eggs when treated with *P. alliacea*, which is a response to the hypothesis proposed in the study, the use of plants as an alternative for anthelmintic treatment.
Hartmann et al., 2018 [22]	Pre-clinical in vitro study (mosquito larvae)	Leaves	Percolation method; Solvents: water and ethanol (hydroalcoholic solution)	Not investigated	1%, 3%, 5%, 10%, 25%, and 50% *v*/*v*.	Not applicable	Anti-dengue study	In this study, the authors evaluated the larvicidal capacity of *P. alliacea*. The authors report that in this study, the cytotoxic effect of *P. alliacea* on *Aedes aegypti* larvae was confirmed, which proves its larvicidal activity.
Lateef et al., 2018 [21]	Pre-clinical in vitro study (microorganisms and enzymatic assays)	Leaves	Infusion method; Solvent: water	Not investigated	5, 8, 10, 12, and 15 μg/mL (Antibacterial assay); 100 and 150 μg/mL (Antifungal assay); 100 μg/mL (Anticoagulant assay); 5, 10, 20 and 40 μg/mL (DPPH assay); 1, 2, 5, 10, and 15 μg/mL (Hydrogen peroxide scavenging activity)	Not applicable	Antimicrobial and anticoagulant study	In this study, the authors investigated the synthesis of silver nanoparticles using *P. alliacea*. The authors report that the described model showed 100% inhibition against *Klebsiella pneumoniae*, *Escherichia coli*, and *Staphylococcus aureus*, as well as antifungal activity and prevented human blood clotting, which confirms the antimicrobial and anticoagulant activities of *P. alliacea.*
Zavala-Ocampo et al., 2022 [2]	Pre-clinical in vitro study (enzymatic assay and human neuroblastoma cell line SH-SY5Y)	Leaves	Maceration method; Solvent: methanol. Fractionation process with methanol, hexane, and ethyl acetate	Methanolic extract: Not investigated; Hexanic fraction: Not investigated; Ethyl acetate fraction: Not investigated; Methanolic extract: Capreoside, Narcissin, Indane, (-)Isocaryophyllene, (-)β -Pinene, €-Tagetone, Peonidin 3-O-sambu -, bioside 5-O-glucoside	Concentrations including 1, 25, 50, 100, 250, 500, 1000, and 2000 μg/mL for the methanolic extract and fractions (Acetylcholinesterase activity); Concentrations including 25, 50, 125, 250, and 500 or the methanolic extract and fractions; Concentrations including 500, 1000, and 2000 μg/mL (Cellular Viability assay) and 1000 μg/mL (Antioxidant activity)	Not applicable	Antioxidant and cognitive study	In this study, the authors investigated extracts, fractions, subfractions, and isolated compounds from *P. alliacea* to study the antioxidant activity and the inhibition of acetylcholinesterase. The authors report in this work that *P. alliacea* has antioxidant properties and demonstrated activity in inhibiting acetylcholinesterase. They also emphasize the importance of investigations in animal models that further explore the therapeutic potential of the species.
Cal et al., 2022 [6]	Pre-clinical in vitro study (cytotoxicity)	Leaves	Maceration method; Solvent: ethanol. Fractionation process with n-hexane, dichloromethane, ethyl acetate, and n-butanol	Protocatechuic acid, cinnamic or benzoic acids, catechin, and epicatechin	5, 10, 15, 20, 25, 50, and 100 μg/mL	Not applicable	Cytotoxicity study	In this study, the authors assessed the potential cytotoxic, genotoxic, and mutagenic effects of ethanolic extract of *P. alliacea* on *Saccharomyces cerevisiae* strains. The results indicate that fractions of mid-polarity of the ethanolic extract at the studied concentrations can induce mutagenicity mediated by oxidative lesions in the mitochondrial and genomic genomes. The authors also state that the lesions caused by the fractions of *P. alliacea* ethanolic extract can be mediated by reactive oxygen species and can reach multiple molecular targets to exert their toxicity.
Prada et al., 2023 [17]	Pre-clinical in vitro study (cytotoxicity)	Leaves	Percolation method; Solvent: ethanol	Myricetin and dibenzyl disulfide	0–500 µg/mL	Not applicable	Cytotoxicity study	In this study, the authors investigated the cytotoxic action of the ethanolic extract of *Petiveria alliacea* against Leukemic cell lines and normal cells originating from the medulla. The results showed that the extract exerted a selective cytotoxic action on leukemic cells, not harming normal marrow cells.
Ex vivo study								
Ballesteros-Ramirez et al., 2020 [10]	Pre-clinical ex vivo study (human leukemic cell line K562)	Leaves	Supercritical fluid extraction (SFE); Solvents: Ethyl acetate, hexane, and methanol. Fractionation process with hexane, chloroform, and methanol solution. According to the authors, three fractions were obtained (FA, FB, and FC). Only FB was used for phytochemical characterization and biological assays	Myricetin and Dibenzyl disulfide	Antioxidant activity tests (ORAC, FRAP, and ABTS assays) and cytotoxicity were performed using a FB stock solution at 250 μg/L	Not applicable	Cytotoxicity study	In this study, the authors evaluated blasts from patients with acute leukemia to analyze the sensitivity of these cells to induction chemotherapy in an ex vivo model. In the work, *P. alliacea* is used to verify if there is a modulation of the sensitivity of these cells to death. The authors state that *P. alliacea* demonstrated positive results in inducing cell death and reinforcing the importance of tests in an ex vivo model to predict treatments, including herbal medicines.
In vitro/in vivo study								
Hoyos et al., 1992 [58]	Pre-clinical in vitro (human lymphocytes) and in vivo study (male mice)	Not applicable (capsules were purchased)	Capsules of *P. alliacea* were mixed with ethanol. After evaporation, the extract was redissolved with dimethyl sulfoxide	Not investigated	1–1000 μg/mL (in vitro assay); 0.51, 102, 153, and 204 mg/kg (in vivo assay)	Not applicable	Toxicological study (genotoxicity)	In this study, the authors investigated the genotoxic effects of *P. alliacea* extract by conducting a sister chromatid exchange method in vitro and in vivo. The authors stated that the plant contains mutagenic and potentially carcinogenic agents. The authors also emphasized that further studies with cells from exposed individuals and experimental animals should be conducted to provide a better evaluation of health risks from the use of the drug.
Williams et al., 1999 [57]	Pre-clinical in vitro (microorganisms) and in vivo study (male mice)	Leaves and stems	Maceration method; Solvents: hexane; Fractionation process with cyclohexane and chloroform	Not investigated	1 mg/mL (in vitro assay); 11 and 23 mg/kg (in vivo assay)	Parenteral administration (Intraperitoneal); Twice weekly for 3 weeks	Immunomodulatory study	In this study, the authors aimed to isolate and characterize the molecules responsible for the immunomodulatory properties of *P. alliacea*. The authors assayed in vitro phagocytosis, in vivo organ assessment, and isolation of dibenzyltrisulfide. The authors stated that *P. alliacea* extract and its isolated compound caused an alteration in blood cell count and an increase in the weight of the thymus and Peyer’s patches, suggesting an increase in the cellular and endocrine process responsible for T-cell differentiation. The authors emphasized that further studies were necessary to elucidate the appropriate mechanisms.
Berger et al., 1998 [55]	Pre-clinical in vitro (bacteria and *T. cruzi*) and in vivo study (mice)	Roots	Percolation method; Solvents: ethanol 95%, n-hexane and methanol. Infusion method; Solvent: water	Not investigated	0.5% (in vitro assays); 100, 10, and 1 mg/kg (in vivo assay)	Oral administration (gavage); Intermittent regimen (every 48 h) for 3 weeks	Antiprotozoal study	In this study, the authors investigated the antiprotozoal activity of extracts from five different plants against *Trypanosoma cruzi* in vitro. Regarding *P. alliacea*, the authors state that the hexane and ethanol extracts of *P. alliacea* leaves and hexane and methanol extracts of the root showed activity against trypomastigotes. However, both plant parts were not effective against epimastigotes. The authors also reported that leaf extracts showed no toxicity in vitro, whereas hexane and methanol extracts were toxic. The authors conclude that the chloroform fraction was very active against trypomastigotes and showed little activity against epimastigotes but showed in vitro toxicity.
Cáceres et al., 1998 [54]	Pre-clinical in vitro (microorganisms) and in vivo study (male mice)	Leaves	Maceration method; Solvents: dichloromethane, ethanol, and water	Not investigated	10 mg/mL (antibacterial and antifungal activity); 1 mg/mL (antitrypanosomal); 10–1000 ppm (cytotoxicity); 500 mg/kg (oral toxicity)	Oral administration (gavage); Intermittent regimen (every 48 h) for 3 weeks	Antimicrobial study	In this study, the authors screened 13 different native plants used in Guatemala for the treatment of protozoal infections, assessing antibacterial and antifungal activities. Regarding *P. alliacea*, the authors state that the extract showed antitrypanosomal activity in vitro but not in vivo. In the toxicity assay, no toxic effects were present in the studied dose (500 mg/kg).
Quadros et al., 1999 [53]	Pre-clinical in vitro study (microorganisms) and in vivo study (male mice)	Roots	Maceration method; Solvents: ethanol and water	Not investigated	1000 mg/kg	Oral administration (gavage); Daily for 5 days, once a day	Immunomodulatory study	In this study, the authors investigated the effects of *Petiveria alliacea* on the hematopoietic response of mice infected with *Listeria monocytogenes*. The authors state that there was a protective effect of the crude extract of *P. alliacea* since the survival of the treated infected was higher than that in the infected group. The authors also suggest an immunomodulation effect of *Petiveria alliacea* extract on hematopoiesis, which may be responsible for the increased resistance of mice to *Listeria monocytogenes* infection.
Hernandez et al., 2014 [31]	Pre-clinical in vitro study (breast adenocarcinoma cell line) and in vivo study (female mice)	Leaves and stems	Maceration method; Solvent: ethanol (96%). The fractionation process was started with ethyl acetate and later with a methanol and water solution	Benzaldehyde, Leridol, Petiveral, Petiveral 4-ethyl, Pinitol, Dibenzyl disulfide, and Dibenzyl trisulfide	125 to 0.95 μg/mL (cytotoxicity); 29.3, 2.9, and 0.29 μg/mL (clonogenic assays); 29.3, 14.6, and 7.3 μg/mL (membrane potential)	Not applicable	Cytotoxicity study	In this study, the authors evaluated the potential biological activity of a *P. alliacea* fraction and its effects in a model of metastatic breast adenocarcinoma (4T1). The authors report that in the study, the fraction of *P. alliacea* in vitro induced apoptosis of 4T1 cells, as well as reporting that there was regression of primary breast tumors. The authors suggest that the glycolytic pathway contributes to elucidating the action of the fraction in antitumor and antiproliferative activity.
Hernandez et al., 2017 [25]	Pre-clinical in vitro (cancer cell lines) and in vivo study (female mice)	Leaves and stems	Reflux extraction; Solvent: ethanol (96%). The ethanolic extract was partitioned with ethyl acetate, yielding a fraction that, in turn, was extracted with methanol and water, producing a dry extract	Benzaldehyde, Leridol, Petiveral, Myricetin, Petiveral 4-ethyl, Pinitol, Dibenzyl disulfide, and Dibenzyl trisulfide	For the cytotoxicity assay, doses ranged from 250 to 0.95 μg/mL. For the in vivo tumor model, the dose used was 250 mg/kg	Parenteral administration (Intraperitoneal); Twice a week for 56 days	Anticancer study	In this study, the authors evaluated the antitumor capacity of *P. alliacea*. To evaluate one of its traditional uses, a murine model of breast cancer was used. The authors reported that *P. alliacea* promoted a decrease in breast cancer cells in the in vitro and in vivo model; mice that were transplanted with tumor and were treated with *P. alliacea* demonstrated a reduction in primary tumor growth.
Zavala-Ocampo et al., 2017 [24]	Pre-clinical in vitro (*Entamoeba histolytica* trophozoite) and in vivo study (male mice)	Leaves	Maceration method; Solvents: methanol and water. A hexane fraction was obtained from the methanolic extract, which was sub-fractionated into three other subfractions (hexane, methanolic, and ethyl acetate)	Hexane subfractions were selected to isolate Isoarborine	The aqueous and methanolic extracts were tested for antiamebic activity and cell viability assay. In addition, the hexane fraction and its subfractions were also tested. Concentrations of all types of extraction range from 0.1 to 1.8 mg/mL. The isolated substance Isoarborinol was also used for Antiamebic activity and cell viability assay at a concentration ranging from 0.05 to 0.3 mg/kg. The hexane fraction was chosen for acute oral toxicity at a dose of 2000 mg/kg	Oral administration (gavage); Single dose	Antiamoebic and Toxicological study	In this study, the authors carried out an investigation of the antiamebic activity of *P. alliacea* in different extracts and fractions. The authors reported that the extracts and fractions demonstrated antiamebic activity, emphasizing that this result is possibly associated with the presence of an important metabolite called isoarborinol.
Ileke et al., 2021 [9]	Pre-clinical in vitro (mosquito larvae and pups) and in vivo study (adult mosquito and male rat)	Leaves	Maceration method; Solvent: absolute ethanol	Not investigated	For all tests performed (Oviposition deterrent, Larvicidal, Pupicidal, Adulticidal, and Repellency tests), concentrations of 50, 100, 200, 400, and 800 mg/L of the ethanolic extract of *P. alliacea* were used		Antimalarial study	In this study, the authors investigated the potency of *P. alliacea* against mosquitoes of the genus *Anopheles* gambiae in different stages of life. The authors also reported that the use of *P. alliaceae* was highly effective, which strengthens the idea of replacing chemical insecticides and reducing the harmful effects related to the use of these substances.
In vivo/in silico study								
Olajubutu et al., 2022 [19]	Pre-clinical in vivo study (female and male rats) and in silico study	Leaves	Maceration method; Solvent: ethanol. Fractionation process with hexane and water	Ethanolic extract: Not investigated; Aqueous fraction: Not investigated; Hexanic fraction: 2,6-Pyridinedicarboxylic acid, Neophytadiene, 2-Pentadecanone, 6,10,14-trimethyl-, 9,12-Octadecadienoic acid (Z,Z)-, n-Hexadecanoic acid, 4,8,12,16-Tetramethylheptadecan-4-olide, n-Eicosane, Hexadecanoic acid, methyl ester, 9-Octadecenoic acid, (E)-, Octadecanoic acid, 9,12-Octadecadienoic acid (Z,Z)-, methyl ester, Hexadecanoic acid, ethyl ester, Oxirane, tetradecyl-, 13-Docosenamide, (Z), 11-Octadecenoic acid, methyl ester, Methyl stearate, Vitamin E, 1,19-Eicosadiene, and Stigmasterol	Ethanolic extract: 1, 2.5	Topical administration	Anti-inflammatory study	In this study, the authors carried out an investigation of the topical anti-inflammatory activity of *P. alliacea* using the paw edema model. The authors state that there was strong topical anti-inflammatory activity and comment that in the evaluation of the molecular docking of some of the most abundant compounds present in the composition of the constituents, some of them proved to be potential topical anti-inflammatory agents. The study also further recommended in vivo studies with isolated compounds to confirm such activities.
Cruz-Salomon et al., 2022 [8]	Pre-clinical in vivo study (male mice) and in silico study	Leaves	Sonication method; Solvents: methanol, hexane, and water	Methanolic extract: Ethyl palmitate, Phytol, Ethyl linoleate, and Squalene; Hexanoic extract: Butylated hydroxytoluene, Methyl oleate, Eicosane, and Squalene; Aqueous extract: Methyl 14-methylpentadecanoate, Methyl oleate, Bis(2-ethylhexyl) maleate, Octadecyl acetate, and 2,4-Bis(1-phenylethyl)phenol	10, 31.6, 100, and 316 mg/kg (for each type of extract)	Oral administration; Single dose	Antinociceptive study	In this study, the authors evaluated the effects of some *P. alliacea* extracts on the antinociceptive activity through the formalin test and on the toxicity of the extracts through the acute oral toxicity test. The authors related the phytochemical composition of the extracts with positive results in the antinociceptive evaluation, where there was a reduction in pain. They also stated that the extracts proved to be safe, with no risk to health.
Literature review								
Luz et al., 2016 [27]	Literature review	Not applicable	Not applicable	Not applicable	Not applicable	Not applicable	Neuropharmacological study	In this study, the authors reviewed aspects of *P. alliacea*, including a compilation of neuropharmacological information about the plant. The authors reported that, based on their research, *P. alliacea* has demonstrated activity in several central nervous system disorders, including depression, anxiety, memory, pain, and epilepsy.
Castañeda et al., 2022 [12]	Literature review	Not applicable	Not applicable	Not applicable	Not applicable	Not applicable	Neuropsychiatric disorders study	In this study, the authors performed a review with the aim of compiling information on the use of medicinal plants in Mesoamerica as a traditional use for the treatment of CNS disorders. The authors provide information about the traditional use of *P. alliacea* as a treatment for dementia, epilepsy, and some nervous treatments. The study reports some information from pharmacological tests performed on animals, pointing out anticonvulsant and anxiolytic activities and effects on memory.

## Data Availability

All data generated in this review are included in this paper. Further enquiries can be directed to the corresponding author.

## Supplementary Materials

The following supporting information can be downloaded at: https://www.mdpi.com/article/10.3390/plants12183343/s1, Table S1: List of excluded articles.
