# Supplementary material for: Amazonian Plants: A Global Bibliometric Approach to Petiveria alliacea L. Pharmacological and Toxicological Properties
Source: Plants (Basel). 2023 Sep 21;12(18):3343. doi: 10.3390/plants12183343 (PMC10536944; doi:10.3390/plants12183343)
Supplement: Supplementary file 1 [file plants-12-03343-s001.zip › plants-2582490-supplementary.pdf]

SUPPLEMENTARY INFORMATION

Amazonian Plants: A global Bibliometric Approach on Pharmacological and Toxicological Properties of *Petiveria alliacea* L.

Brenda Costa da Conceição[1], Thales Andrade da Silva[1], Lucas Villar Pedrosa da Silva Pantoja[1], Diandra Araújo Luz[1], Eloise Karolina Serrão Cardoso[1], Laryssa Danielle da Silva Reis[1], Maria Carolina Raiol da Silva[1], Monique Silva Kussler[1], Cristiane do Socorro Ferraz Maia[1], Enéas de Andrade Fontes-Júnior[1,*]

.

1 Laboratory of Inflammation and Behavioral Pharmacology, Health Science Institute, Federal University of Pará, Belém-PA, Brazil.

* *Corresponding author.*

CONTENTS

Page 2. Supplementary Information1. List of excluded articles.

Authors	Article Title	Journal	WoS-CC citations	Reason for exclusion
Alvarez, NH; Stegmayer, MI; Seimandi, GM; Pensiero, JF; Zabala, JM; Favaro, MA; Derita, MG	Natural Products Obtained from Argentinean Native Plants Are Fungicidal Against Citrus Postharvest Diseases	Horticulturae	0	Focus on Agricultural Sciences
Herrera-Gorocica, AM; Ruiz-Sanchez, E; Ballina-Gomez, HS; Reyes-Solis, G; Sanchez-Lazaro, A	Response of Bemisia Tabaci Genn To the Association Tomato-Aromatic Plant	Agrociencia	0	Focus on Agricultural Sciences
Wooten, J; Mavingire, N; Araujo, CA; Aja, J; Wauchope, S; Delgoda, R; Brantley, E	Dibenzyl Trisulfide Induces Caspase-Independent Death and Lysosomal Membrane Permeabilization Of Triple-Negative Breast Cancer Cells	Fitoterapia	1	Focus on *Petiveria alliacea* secondary metabolites
Clarke, N; Irvine, W	*In Silico* Design and SAR Study of Dibenzyl Trisulfide Analogues for Improved CYP1A1 Inhibition	Chemistryopen	1	Focus on *Petiveria alliacea* secondary metabolites
Olajubutu, OG; Ogunremi, BI; Adewole, AH; Awotuya, OI; Fakola, EG; Anyim, G; Faloye, KO	Correction To: Topical Anti-Inflammatory Activity of *Petiveria alliacea*, Chemical Profiling and Computational Investigation of Phytoconstituents Identified from its Active Fraction	Chemistry Africa	0	Correction letter
Lopez, JJ; Chirinos, DT; Ponce, WH; Solorzano, RF; Alarcon, JP	Insecticide Activity of Botanical Formulates on The Fall Armyworm, *Spodoptera frugiperda* (Lepidoptera: Noctuidae)	Revista Colombiana de Entomologia	0	Focus on Agricultural Sciences
Kujawska, M; Schmeda-Hirschmann, G	The Use of Medicinal Plants by Paraguayan Migrants in The Atlantic Forest of Misiones, Argentina, Is Based on Guarani Tradition, Colonial and Current Plant Knowledge	Journal of Ethnopharmacology	8	Ethnopharmacological study

Wauchope, S; Roy, MA; Irvine, W; Morrison, I; Brantley, E; Gossell-Williams, M; Timme-Laragy, AR; Delgoda, R	Dibenzyl Trisulfide Binds to And Competitively Inhibits the Cytochrome P450 1A1 Active Site Without Impacting the Expression of The Aryl Hydrocarbon Receptor	Toxicology and Applied Pharmacology	3	Focus on *Petiveria alliacea* secondary metabolites
Flota-Burgos, GJ; Rosado-Aguilar, JA; Rodriguez-Vivas, RI; Borges-Argaez, R; Martinez-Ortiz-de-Montellano, C; Gamboa-Angulo, M	Anthelmintic Activity of Extracts and Active Compounds from diospyros *Anisandraonancylostoma caninum*, *Haemonchus placeiand Cyathostomins*	Frontiers in Veterinary Science	7	*Petiveria alliacea* is not the focus of the paper
Pettinelli, JD; Soares, BD; Collin, M; Mansur, EA; Engelmann, F; Gagliardi, RF	Cryotolerance Of Somatic Embryos of Guinea (*Petiveria Alliacea*) To V-Cryoplate Technique and Histological Analysis of Their Structural Integrity	Acta Physiologiae Plantarum	2	Plant anatomy study
Figueroa Gualteros, AM; Castro Trivino, EA; Castro Salazar, HT	Bioplaguicide Effect of Vegetal Extracts to Control of *Spodoptera frugiperda* in Corn Crop (*Zea mays*)	Acta Biologica Colombiana	3	Focus on Agricultural Sciences
He, TY; Chambers, MI; Musah, RA	Application of Direct Analysis in Real Time-High Resolution Mass Spectrometry to Investigations of Induced Plant Chemical Defense Mechanisms-Revelation of Negative Feedback Inhibition of An Alliinase	Analytical Chemistry	3	Plant physiology study
Violeth, JLB; Herrera, CF; Garcia, KDP	Plant Extracts: Alternative Control *Colaspis sp*. (Coleoptera: Chrysomelidae) In Banana Cv. Harton	Temas Agrarios	1	Focus on Agricultural Sciences
Drane, E; Feliot-Rippeault, M; Smith-Ravin, J; Marcelin, O	Ethnobotanical Study in Martinique of the Species Behind the Local Plant Name Bwa Kaka	Ethnobiology Letters	1	Ethnopharmacological study
He, TY; Misuraca, JC; Musah, RA	Carboranyl-Cysteine-Synthesis, Structure and Self-Assembly Behavior of a Novel Alpha-Amino Acid	Scientific Reports	16	Chemical synthesis study

de Melo, KA; Carrenho, R	Growth and Mycorrhizal Colonization of Maize Plants Treated with Aqueous Extracts from Nonmycorrhizal Weeds	Pesquisa Agropecuaria Brasileira	0	Focus on Agricultural Sciences
Foster, K; Younger, N; Aiken, W; Brady-West, D; Delgoda, R	Reliance on Medicinal Plant Therapy Among Cancer Patients in Jamaica	Cancer causes & Control	22	Ethnopharmacological study
Silvaroli, JA; Pleshinger, MJ; Banerjee, S; Kiser, PD; Golczak, M	Enzyme That Makes You Cry-Crystal Structure of Lachrymatory Factor Synthase from *Allium cepa*	Acs Chemical Biology	13	*Petiveria alliacea* is not the focus of the paper
Pettinelli, JD; Soares, BD; Cantelmo, L; Garcia, RD; Mansur, E; Engelmann, F; Gagliardi, RF	Cryopreservation of Somatic Embryos from *Petiveria alliacea* L. By Different Techniques Based on Vitrification	In Vitro Cellular & Developmental Biology-Plant	6	Plant physiology study
Pagani, E; Santos, JDL; Rodrigues, E	Culture-Bound Syndromes of a Brazilian Amazon Riverine Population: Tentative Correspondence Between Traditional and Conventional Medicine Terms and Possible Ethnopharmacological Implications	Journal of Ethnopharmacology	8	Ethnopharmacological study
Sershen; Mdamba, B; Ramdhani, S	Propagule and Seedling Responses of Three Species Naturalised In Subtropical South Africa To Elevated Temperatures	Flora	0	Plant physiology study
Garcia-Perez, ME; Lemus-Rodriguez, Z; Hung-Arbelo, M; Vistel-Vigo, M	Influence of Polyvinylpyrrolidone, Microcrystalline Cellulose and Colloidal Silicon Dioxide on Technological Characteristics of a High-Dose *Petiveria alliacea* Tablet	Drug Development and Industrial Pharmacy	0	Pharmaceutical technology study
Molina, CM; Pringle, JK; Saumett, M; Evans, GT	Geophysical and Botanical Monitoring of Simulated Graves in a Tropical Rainforest, Colombia, South America	Journal Of Applied Geophysics	11	Geophysical study

Arceo-Medina, GN; Rosado-Aguilar, JA; Rodriguez-Vivas, RI; Borges-Argaez, R	Synergistic Action of Fatty Acids, Sulphides and Stilbene Against Acaricide-Resistant Rhipicephalus Microplus Ticks	Veterinary Parasitology	11	Focus on *Petiveria alliacea* secondary metabolites
Kasper, SH; Bonocora, RP; Wade, JT; Musah, RA; Cady, NC	Chemical Inhibition of Kynureninase Reduces Pseudomonas Aeruginosa Quorum Sensing and Virulence Factor Expression	Acs Chemical Biology	28	Focus on *Petiveria alliacea* secondary metabolites
Oliveira, GL; Oliveira, AFM; Andrade, LDC	Medicinal and Toxic Plants from Muribeca Alternative Health Center (Pernambuco, Brazil): An Ethnopharmacology Survey	Boletin Latinoamericano Y Del Caribe De Plantas Medicinales Y Aromaticas	2	Ethnopharmacological study
Lim, J; Pyun, J; Char, K	Recent Approaches for The Direct Use of Elemental Sulfur in The Synthesis and Processing of Advanced Materials	Angewandte Chemie-International Edition	189	Chemical synthesis study
Granados-Echegoyen, C; Perez-Pacheco, R; Bautista-Martinez, N; Alonso-Hernandez, N; Sanchez-Garcia, JA; Martinez-Tomas, SH; Sanchez-Mendoza, S	Insecticidal Effect of Botanical Extracts on Developmental Stages of *Bactericera cockerelli* (Sulc) (Hemiptera: Triozidae)	Southwestern Entomologist	8	Bioplaguicide study
Kuo, PC; Thang, TD; Huang, GJ; Huang, BS; Hoa, LTM; Yang, ML; Wu, TS	Flavonoids from The Fruits of *Desmos cochinchinesis* Var. Fulvecens and Their Inhibitory Effects on no Production	Chemistry Of Natural Compounds	3	*Petiveria alliacea* is not the focus of the paper
Kasper, SH; Samarian, D; Jadhav, AP; Rickard, AH; Musah, RA; Cady, NC	S-Aryl-L-Cysteine Sulphoxides and Related Organosulphur Compounds Alter Oral Biofilm Development And AI-2-Based Cell-Cell Communication	Journal Of Applied Microbiology	10	Focus on *Petiveria alliacea* secondary metabolites
Kerdudo, A; Gonot, V; Ellong, EN; Rochefort, K; Boyer, L; Michel, T; Fernandez, X	New Antibacterial Compounds from Plant Biodiversity	Planta Medica	1	Meeting Abstract

Patra, JK; Kim, ES; Oh, K; Kim, HJ; Kim, Y; Baek, KH	Antibacterial Effect of Crude Extract and Metabolites of *Phytolacca americana* on Pathogens Responsible for Periodontal Inflammatory Diseases and Dental Caries	BMC Complementary and Alternative Medicine	34	*Petiveria alliacea* is not the focus of the paper
Lowe, HIC; Facey, COB; Toyang, NJ; Bryant, JL	Specific RSK Kinase Inhibition by Dibenzyl Trisulfide and Implication for Therapeutic Treatment of Cancer	Anticancer Research	10	Focus on *Petiveria alliacea* secondary metabolites
Castellar, A; Gagliardi, RF; Mansur, E; Bizzo, HR; Souza, AM; Leitao, SG	Volatile Constituents from *in vitro* and *ex vitro* Plants of *Petiveria alliacea* L.	Journal of Essential Oil Research	7	Phytochemical study
Leal, CM; Orozco, N; Rivera, J; Monterroso, L; Caceres, A	*In Vitro* Activity Against Helicobacter Pylori by Ethanol Extracts from Sixteen Medicinal Plants Used for Chronic Gastrointestinal Diseases in Guatemala	International Symposium on Medicinal Plants and Natural Product S	3	Proceedings Paper
Lester, DA; Richards, AA; Younger-Coleman, NO; Pepple, DJ	Cilostazol And Blood Viscoelasticity in Homozygous Sickle Cell Disease	British Journal of Biomedical Science	1	*Petiveria alliacea* is not the focus of the paper
Cantelmo, L; Soares, BO; Rocha, LP; Pettinelli, JA; Callado, CH; Mansur, E; Castellar, A; Gagliardi, RF	Repetitive Somatic Embryogenesis from Leaves of The Medicinal Plant *Petiveria alliacea* L.	Plant Cell Tissue and Organ Culture	14	Plant physiology study
Souza, CR; Guedes, RC; Fusco-Almeida, AM; Oliveira, WP	Spray Dried *Petiveria alliacea* Extracts: Standardization and Evaluation of The Antimicrobial Activity	Planta Medica	0	Meeting Abstract
Mitchell, SA	Ethnomedicinal Opportunities for Human Health from Jamaican-Grown Tropical Herbs: Anti-Cancer Guinea Hen Weed (*Petiveria alliacea*), Bitter Herbs, Root Tonics, Nurtaceuticals, Spices and Cold Bushes.	*In Vitro* Cellular & Developmental Biology-Animal	0	Ethnopharmacological study

Soares, BO; Fernandes, DC; Cantelmo, L; Rocha, LP; Pettinelli, JA; Christo, AG; Coelho, MGP; Gagliardi, RF	Botanical Characterization of *Petiveria alliacea* L. from Rio De Janeiro, Brazil: Systematic and Functional Implications	Plant Biosystems	4	Botanical study
Joyce, NI; Eady, CC; Silcock, P; Perry, NB; van Klink, JW	Fast Phenotyping Of LFS-Silenced (Tearless) Onions by Desorption Electrospray Ionization Mass Spectrometry (DESI-MS)	Journal of Agricultural and Food Chemistry	39	Focus on Agricultural Sciences
Kubec, R; Krejcova, P; Mansur, L; Garcia, N	Flavor Precursors and Sensory-Active Sulfur Compounds in Alliaceae Species Native to South Africa And South America	Journal of Agricultural and Food Chemistry	22	Focus on Agricultural Sciences
Cruz-Estrada, A; Gamboa-Angulo, M; Borges-Argaez, R; Ruiz-Sanchez, E	Insecticidal Effects of Plant Extracts on Immature Whitefly *Bemisia tabaci* Genn. (Hemiptera: Aleyroideae)	Electronic Journal Of Biotechnology	14	Bioplaguicide study
Ding, LJ; Ding, W; Zhang, YQ; Luo, JX	Bioguided Fractionation and Isolation of Esculentoside P from *Phytolacca americana* L.	Industrial Crops And Products	21	*Petiveria alliacea* is not the focus of the paper
Zheng, F; Pratt, DA	Antioxidant Generation and Regeneration in Lipid Bilayers: The Amazing Case of Lipophilic Thiosulfinates And Hydrophilic Thiols	Chemical Communications	9	*Petiveria alliacea* is not the focus of the paper
Freeman, F; Bui, A; Dinh, L; Hehre, WJ	Dehydrative Cyclocondensation Mechanisms of Hydrogen Thioperoxide And of Alkanesulfenic Acids	Journal of Physical Chemistry A	7	*Petiveria alliacea* is not the focus of the paper
Cruz, LM; Vera, J; Melendez, E; Rivera-Portalatin, N	Cytotoxic Studies of The Essential Oils of *Petiveria aliacea* In Colon Cancer Cells	Planta Medica	1	Meeting Abstract
Cady, NC; McKean, KA; Behnke, J; Kubec, R; Mosier, AP; Kasper, SH; Burz, DS; Musah, RA	Inhibition of Biofilm Formation, Quorum Sensing and Infection in Pseudomonas Aeruginosa by Natural Products-Inspired Organosulfur Compounds	Plos One	140	Focus on *Petiveria alliacea* secondary metabolites

Christie, SLF; Levy, ASA	Investigation of Hypoglycemic Activity of Four Extracts of *Petiveria alliacea* (Guinea Hen Weed) In Normoglycemic Experimental Rat Models	Faseb Journal	0	Meeting Abstract
Masamura, N; Ohashi, W; Tsuge, N; Imai, S; Ishii-Nakamura, A; Hirota, H; Nagata, T; Kumagai, H	Identification of Amino Acid Residues Essential for Onion Lachrymatory Factor Synthase Activity	Bioscience Biotechnology And Biochemistry	6	*Petiveria alliacea* is not the focus of the paper
Nwachukwu, ID; Slusarenko, AJ; Gruhlke, MCH	Sulfur and Sulfur Compounds in Plant Defence	Natural Product Communications	31	Focus on *Petiveria alliacea* secondary metabolites
Santander, SP; Hernandez, JF; Barreto, CC; Masayuki, A; Moins-Teisserenc, H; Fiorentino, S	Immunomodulatory Effects of Aqueous and Organic Fractions from *Petiveria Alliacea* On Human Dendritic Cells (Vol 40, Pg 833, 2012)	American Journal Of Chinese Medicine	0	Correction letter
He, Q; Kubec, R; Jadhav, AP; Musah, RA	First Insights into The Mode of Action of a Lachrymatory Factor Synthase - Implications for The Mechanism of Lachrymator Formation in *Petiveria alliacea*, *Allium cepa* And Nectaroscordum Species	Phytochemistry	11	Plant physiology study
Illnait-Zaragozi, MT; Velar-Martinez, RE; Martinez-Machin, GF; Andreu, CMF; Perurena-Lancha, MR; Illnait-Ferrer, J; Garcia, AB; Meis, JF	Antifungal Effects of a Hydroalcoholic Extract of *Petiveria alliacea* L on Clinical *Candida spp*.	Mycoses	0	Meeting Abstract
Sahebkar, A; Iranshahi, M	Volatile Constituents of The *Genus ferula* (Apiaceae): A Review	Journal of Essential Oil-Bearing Plants	50	*Petiveria alliacea* is not the focus of the paper
Druga, B; Suteu, D; Rosca-Casian, O; Parvu, M; Dragos, N	Two Novel *Alliin lyase* (Alliinase) Genes from Twisted-Leaf Garlic (*Allium obliquum*) and Mountain Garlic (*Allium senescens* Var. Montanum)	Notulae Botanicae Horti Agrobotanici Cluj-Napoca	5	*Petiveria alliacea* is not the focus of the paper
Castellar, A; Gagliardi, RF; Mansur, E	*In Vitro* Propagation and Establishment of Callus and Cell Suspension Cultures of *Petiveria alliacea* L., A Valuable Medicinal Plant	Journal of Medicinal Plants Research	4	Plant physiology study

Baez, C; Rodriguez, F; Ospina, CA; Pagan, M	Biological Evaluation and Chemical Analysis of Extracts from *Petiveria alliacea*	Abstracts of Papers of The American Chemical Society	0	Meeting Abstract
Amaral, ACF; Silva, JRD; Falcao, DQ; Ferreirinha, LG; dos Santos, AR; Araujo, RB; Ferreira, JLP	Chemical Analysis of Toxic Principles in Preparations of *Ruta graveolens* And *Petiveria alliacea*	Poisoning by Plants, Mycotoxins, And Related Toxins	0	Phytochemical study
Lynett, PT; Butts, K; Vaidya, V; Garrett, GE; Pratt, DA	The Mechanism of Radical-Trapping Antioxidant Activity of Plant-Derived Thiosulfinates	Organic & Biomolecular Chemistry	41	Focus on *Petiveria alliacea* secondary metabolites
Neves, ID; da Camara, CAG; de Oliviera, JCS; de Almeida, AV	Acaricidal Activity and Essential Oil Composition of *Petiveria alliacea* L. From Pernambuco (Northeast Brazil)	Journal of Essential Oil Research	12	Bioplaguicide study
Mayorga, P; Perez, KR; Cruz, SM; Caceres, A	Comparison of Bioassays Using the Anostracan Crustaceans Artemia Salina and *Thamnocephalus platyurus* For Plant Extract Toxicity Screening	Revista Brasileira de Farmacognosia-Brazilian Journal of Pharmacognosy	24	Bioplaguicide study
Pepple, DJ; Richards, AA; Lowe, DA; Reid, WA; Younger, NO; Williams, LAD	*In Vitro* Erythrocytic Membrane Effects of Dibenzyl Trisulfide, A Secondary Metabolite of *Petiveria alliacea*	Fitoterapia	5	Focus on *Petiveria alliacea* secondary metabolites
Sahebkar, A; Iranshahi, M	Biological Activities of Essential Oils from The *Genus Ferula* (Apiaceae)	Asian Biomedicine	65	*Petiveria alliacea* is not the focus of the paper
de Oliveira, GL; de Oliveira, AFM; Andrade, LDC	Medicinal Plants Used in The Urban Community of Muribeca, Northeast Brazil	Acta Botanica Brasilica	27	Ethnopharmacological study
Rosado-Aguilar, JA; Aguilar-Caballero, A; Rodriguez-Vivas, RI; Borges-Argaez, R; Garcia-Vazquez, Z; Mendez-Gonzalez, M	Acaricidal Activity of Extracts from *Petiveria alliacea* (Phytolaccaceae) Against the Cattle Tick, Rhipicephalus (Boophilus) Microplus (Acari: Ixodidae)	Veterinary Parasitology	70	Bioplaguicide study

Martinez, GJ	Natural Remedies in The Prevention and Oral Health Care of The Toba From Central Chaco (Argentina)	Boletin Latinoamericano Y Del Caribe De Plantas Medicinales Y Aromaticas	10	Ethnopharmacological study
Kubec, R; Cody, RB; Dane, AJ; Musah, RA; Schraml, J; Vattekkatte, A; Block, E	Applications of Direct Analysis in Real Time-Mass Spectrometry (DART-MS) In Allium Chemistry. (Z)-Butanethial S-Oxide And 1-Butenyl Thiosulfinates And Their S-(E)-1-Butenylcysteine S-Oxide Precursor from *Allium siculum*	Journal of Agricultural and Food Chemistry	72	Chemical synthesis study
Xu, WH; Xi, B; Wu, JY; An, HY; Zhu, J; Abassi, Y; Feinstein, SC; Gaylord, M; Geng, BQ; Yan, HF; Fan, WM; Sui, MH; Wang, XB; Xu, X	Natural Product Derivative Bis(4-Fluorobenzyl) Trisulfide Inhibits Tumor Growth by Modification of Beta-Tubulin at Cys 12 and Suppression of Microtubule Dynamics	Molecular Cancer Therapeutics	20	Focus on *Petiveria alliacea* secondary metabolites
Musah, RA; He, Q; Kubec, R	Discovery and Characterization of a Novel Lachrymatory Factor Synthase in *Petiveria alliacea* and its Influence on Alliinase-Mediated Formation of Biologically Active Organosulfur Compounds	Plant Physiology	27	Plant physiology study
Musah, RA; He, Q; Kubec, R; Jadhav, A	Studies of a Novel Cysteine Sulfoxide Lyase From *Petiveria alliacea*: The First Heteromeric Alliinase	Plant Physiology	15	Focus on *Petiveria alliacea* secondary metabolites
Williams, LAD; Barton, EN; Kraus, W; Rosner, H	Implications of Dibenzyl Trisulphide For Disease Treatment Based on Its Mode of Action	West Indian Medical Journal	6	Focus on *Petiveria alliacea* secondary metabolites
Singh, SP; Lough, AJ; Schwan, AL	Bis(2-Bromobenzyl) Trisulfide	Acta Crystallographica Section E-Crystallographic Communications	0	Chemical synthesis study

Gardiano, CG; Ferraz, S; Lopes, EA; Ferreira, PA; Amora, DX; de Freitas, LG	Evaluation of Plant Aqueous Extracts, Added into The Soil, on Meloidogyne Javanica (Treub, 1885) Chitwood, 1949	Semina-Ciencias agrarias	10	Focus on Agricultural Sciences
Illnait-Zaragozi, MT; Illnait-Ferrer, J; Garcia, AB	Evaluation of The Antimycotic Effect of *Petiveria alliacea* L	Mycoses	0	Meeting Abstract
Bao, YM; Mo, XP; Xu, XY; He, YY; Xu, X; An, HY	Stability Studies of Anticancer Agent Bis(4-Fluorobenzyl) Trisulfide And Synthesis of Related Substances	Journal of Pharmaceutical and Biomedical Analysis	14	Focus on *Petiveria alliacea* secondary metabolites
Rodrigues, E; Gianfratti, B; Tabach, R; Negri, G; Mendes, FR	Preliminary Investigation of The Central Nervous System Effects of 'Tira-Capeta' (Removing the Devil), A Cigarette Used by Some Quilombolas Living in Pantanal Wetlands of Brazil	Phytotherapy Research	8	Ethnopharmacological study
Webster, SA; Mitchell, SA; Gallimore, WA; Williams, LAD; Ahmad, MH	Biosynthesis of Dibenzyl Trisulfide (DTS) From Somatic Embryos and Rhizogenous/Embryogenic Callus Derived from Guinea Hen Weed (*Petiveria alliacea* L.) Leaf Explants	In Vitro Cellular & Developmental Biology-Plant	8	Focus on *Petiveria alliacea* secondary metabolites
Iranshahi, M; Hassanzadeh-Khayat, M; Bazzaz, BSF; Sabeti, Z; Enayati, F	High Content of Polysulphides In the Volatile Oil of *Ferula latisecta* Rech. R. Et Aell. Fruits and Antimicrobial Activity of The Oil	Journal of Essential Oil Research	31	Focus on *Petiveria alliacea* secondary metabolites
Okada, Y; Tanaka, K; Sato, E; Okajima, H	Antioxidant Activity of the New Thiosulfinate Derivative, S-Benzyl Phenylmethanethiosulfinate, From *Petiveria Alliacea* L.	Organic & Biomolecular Chemistry	25	Focus on *Petiveria alliacea* secondary metabolites
Garcia-Mateos, MR; Sanchez, EE; Espinosa-Robles, P; Alvarez-Sanchez, ME	Toxicity of *Petiveria alliacea* L. On Greenhouse Whitefly (*Trialeurodes vaporariorum* WEST.)	Interciencia	8	Bioplaguicide study

Mitchell, SA; Ahmad, MH	Medicinal Plant Biotechnology Research in Jamaica - Challenges and Opportunities	Proceedings of The International Symposium on Medicinal and Nutraceutical Plants	2	Biotechnology study
Williams, LAD; Rosner, H; Levy, H; Barton, EN	A Critical Review of The Therapeutic Potential of Dibenzyl Trisulphide Isolated from *Petiveria alliacea* L (Guinea Hen Weed, Anamu)	West Indian Medical Journal	35	Focus on *Petiveria alliacea* secondary metabolites
An, HY; Zhu, J; Wang, XB; Xu, X	Synthesis and Anti-Tumor Evaluation of New Trisulfide Derivatives	Bioorganic & Medicinal Chemistry Letters	28	Focus on *Petiveria alliacea* secondary metabolites
Mitchell, SA; Ahmad, MH	A Review of Medicinal Plant Research at The University of The West Indies, Jamaica, 1948-2001	West Indian Medical Journal	60	Ethnopharmacological study
Kim, S; Kubec, R; Musah, RA	Antibacterial and Antifungal Activity of Sulfur-Containing Compounds from *Petiveria alliacea* L.	Journal of Ethnopharmacology	94	Focus on *Petiveria alliacea* secondary metabolites
Caceres, A; Gupta, MP; Ocampo, RA; Mendoza, J; Herrera, MR; Solis, PN; Cruz, SM; Martinez, V	Multidisciplinary Development of Phytotherapeutic Products from Native Central American Plants	Proceedings of The Fourth International Conference on Quality and Safety Issues Related to Botanicals	3	Proceedings Paper
Lans, CA	Ethnomedicines Used in Trinidad And Tobago For Urinary Problems and Diabetes Mellitus	Journal of Ethnobiology and Ethnomedicine	221	Ethnopharmacological study
Duarte, MR; Lopes, JF	Leaf and Stem Morphoanatomy of *Petiveria alliacea*	Fitoterapia	9	Botanical study
Kubec, R; Musah, RA	Gamma-Glutamyl Dipeptides in *Petiveria alliacea*	Phytochemistry	12	Phytochemical study
Musah, RA; Kim, E; Kubec, R	Antibacterial and Antifungal Activity of Sulfur-Containing Compounds from *Petiveria alliacea* L.	Phosphorus Sulfur and Silicon and the Related Elements	6	Focus on Petiveria alliacea secondary metabolites

Peraza-Sanchez, SR; Chan-Che, EO; Ruiz-Sanchez, E	Screening of Yucatecan Plant Extracts to Control Colletotrichum Gloeosporioides And Isolation of a New Pimarene from *Acacia pennatula*	Journal of Agricultural and Food Chemistry	18	Focus on Agricultural Sciences
Perez-Leal, R; Garcia-Mateos, MR; Vasquez-Rojas, TR; Colinas-Leon, TC	Allelopathic Potential of *Petiveria alliacea* L.	Agronomy for Sustainable Development	7	Focus on Agricultural Sciences
Webster, SA; Mitchell, SA; Ahmad, MH	Somatic Embryogenic Response of *Petiveria alliacea* Leaf Explant-Derived Callus to Sucrose and Glucose Enriched Media Supplemented with NAA and BAP	*In Vitro* Cellular & Developmental Biology-Animal	2	Plant physiology study
Nascimento, C; Moura, A	Effect of *Petiveria alliacea* L. Extracts on Pleural Macrophages and on BCG-Induced Inflammatory Response	Immunology 2004: Genomic Issues, Immune System Activation and Allergy	0	Proceedings Paper
Zuluaga, H; Oviedo, A; Solarte, E; Pena, EJ	Light Stress Effect and By Nitrogen Deficiency in Plants of *Petiveria alliacea* Measured with Two Chlorophyll Fluorescence Technique	Riao/Optilas 2004: 5th Iberoamerican Meeting on Optics And 8th Latin American Meeting on Optics, Lasers, And Their Applications, Pts 1-3: Ico Regional Meeting	0	Proceedings Paper
Williams, LAD; Vasquez, E; Klaiber, I; Kraus, W; Rosner, H	A Sulfonic Anhydride Derivative from Dibenzyl Trisulphide With Agro-Chemical Activities	Chemosphere	12	Focus on Petiveria alliacea secondary metabolites
Kubec, R; Kim, S; Musah, RA	The Lachrymatory Principle of *Petiveria alliacea*	Phytochemistry	31	Plant physiology study
Kubec, R; Kim, S; Musah, RA	S-Substituted Cysteine Derivatives and Thiosulfinate Formation in *Petiveria alliacea* - Part II	Phytochemistry	39	Phytochemical study
Kim, S; Kubec, R; Musah, RA	Anticancer, Antibacterial and Antifungal Activities of Sulfur-Containing Compounds from *Petiveria alliacea*.	Abstracts of Papers of The American Chemical Society	0	Meeting Abstract

Zoghbi, MDB; Andrade, EHA; Maia, JGS	Volatile Constituents from Adenocalymma *Alliaceum miers* And *Petiveria alliacea* L., Two Medicinal Herbs of The Amazon	Flavour And Fragrance Journal	24	Phytochemical study
Kubec, R; Musah, RA	Cysteine Sulfoxide Derivatives in *Petiveria alliacea*	Phytochemistry	54	Focus on Petiveria alliacea secondary metabolites
Cifuentes, CM; Gomez-Serranillos, MP; Iglesias, I; del Fresno, AMV	Neuropharmacological Profile of Ethnomedicinal Plants of Guatemala	Journal of Ethnopharmacology	21	Ethnopharmacological study
Mata-Greenwood, E; Ito, A; Westenburg, H; Cui, BL; Mehta, RG; Kinghorn, AD; Pezzuto, JM	Discovery of Novel Inducers of Cellular Differentiation Using HL-60 Promyelocytic Cells	Anticancer Research	40	Focus on Petiveria alliacea secondary metabolites
Ayodele, ET; Hudson, HR; Ojo, IAO; Pianka, M	Organosulfur Compounds as a Potential Fungicides: The Preparation and Properties of Some Substituted Benzyl 2-Hydroxyethyl Oligosulfides	Phosphorus Sulfur and Silicon and the Related Elements	7	Focus on Petiveria alliacea secondary metabolites
Oluwole, FS; Bolarinwa, AF	The Uterine Contractile Effect of *Petiveria alliacea* Seeds	Fitoterapia	7	Article not found
Johnson, L; Williams, LAD; Roberts, EV	An Insecticidal and Acaricidal Polysulfide Metabolite from The Roots of *Petiveria alliacea*	Pesticide Science	25	Bioplaguicide study
DelleMonache, F; Menichini, F; Suarez, LEC	*Petiveria alliacea* .2. Further Flavonoids and Triterpenes	Gazzetta Chimica Italiana	19	Phytochemical study
Diaz, OD; Portuondo, MC; Valdes, MG; Chang, RR; Handal, E	Comparison of Various Digestion Methods for The Determination of Different Metals in *Petiveria alliacea* Lynn (Anamu) By Flame Absorption Spectrometry	Quimica Analitica	2	Chemical synthesis study
SERTIE, JAA; GERMANO, DHP; SUDO, LS; HANADA, S	*Petiveria alliacea* - Analgesic Effect and Preclinical Toxicity	Journal Of Dental Research	2	Meeting Abstract
SERTIE, JAA; HANADA, S; SUDO, LS; GERMANO, DHP	*Petiveria alliacea* - Antiinflammatory Effect and Gastric Mucous Protection	Journal Of Dental Research	2	Meeting Abstract

MALPEZZI, ELA; DAVINO, SC; COSTA, LV; FREITAS, JC; GIESBRECHT, AM; ROQUE, NF	Antimitotic Action of Extracts of *Petiveria alliacea* On Sea-Urchin Egg Development	Brazilian Journal of Medical and Biological Research	14	Proceedings Paper
DELLEMONACHE, F; SUAREZ, LEC	6-C-Formyl And 6-C-Hydroxymethyl Flavanones from *Petiveria alliacea*	Phytochemistry	27	Phytochemical study
GIRON, LM; FREIRE, V; ALONZO, A; CACERES, A	Ethnobotanical Survey of The Medicinal Flora Used by The Caribs Of Guatemala	Journal of Ethnopharmacology	138	Ethnopharmacological study
DESOUSA, JR; DEMUNER, AJ; PINHEIRO, JA; BREITMAIER, E; CASSELS, BK	Dibenzyl Trisulfide And Trans-N-Methyl-4-Methoxyproline From *Petiveria alliacea*	Phytochemistry	35	Focus on Petiveria alliacea secondary metabolites
Segelman, FP; Segelman, AB	Constituents of *Petiveria alliacea* L (Phytolaccaceae) .1. Isolation of Isoarborinol, Isoarborinol Acetate and Isoarborinol Cinnamate From Leaves	Lloydia-The Journal of Natural Products	5	Meeting Abstract
Adesogan, EK	Trithiolaniacin, A Novel Trithiolan From *Petiveria-alliacea*	Journal of The Chemical Society-Chemical Communications	21	Phytochemical study
Szczepan, CV; Zgorzela, P; Hoyer, GA	Isolation, Structure Elucidation, and Synthesis of an Antimicrobial Substance from *Petiveria-alliacea* L	Arzneimittel-Forschung/Drug Research	6	Chemical synthesis study
